# Enhancing the Green Synthesis of Glycerol Carbonate: Carboxylation of Glycerol with CO_2_ Catalyzed by Metal Nanoparticles Encapsulated in Cerium Metal–Organic Frameworks

**DOI:** 10.3390/nano14080650

**Published:** 2024-04-09

**Authors:** Simon Lukato, Michał Wójcik, Agnieszka Krogul-Sobczak, Grzegorz Litwinienko

**Affiliations:** Faculty of Chemistry, University of Warsaw, Pasteura 1, 02-093 Warsaw, Poland; s.lukato@uw.edu.pl (S.L.);

**Keywords:** glycerol carbonate, carbon dioxide, glycerol, metal nanoparticles, metal organic frameworks, NPs-CeMOFs

## Abstract

The reaction of glycerol with CO_2_ to produce glycerol carbonate was performed successfully in the presence of gold nanoparticles (AuNPs) supported by a metal–organic framework (MOF) constructed from mixed carboxylate (terephthalic acid and 1,3,5-benzenetricarboxylic acid). The most efficient were two AuNPs@MOF catalysts prepared from pre-synthesized MOF impregnated with Au^3+^ salt and subsequently reduced to AuNPs using H_2_ (catalyst 4%Au(H_2_)@MOF1) or reduced with NaBH_4_ (catalyst 4%Au@PEI-MOF1). Compared to existing catalysts, AuNPs@MOFs require simple preparation and operate under mild and sustainable conditions, i.e., a much lower temperature and the lowest CO_2_ overpressure ever reported, with MgCO_3_ having been found to be the optimal dehydrating agent. Although the yield of the process is still not competitive with previously developed systems, the most promising advantage is the highest TOF (78 h^−1^) ever reported for this reaction. The optimal parameters observed for AuNPs were also tested on AgNPs and CuNPs with promising results, suggesting their great potential for industrial application. The catalysts were characterized by XRD, TEM, SEM-EDS, ICP-MS, XPS, and porosity measurements, confirming that AuNPs are present in low concentration, uniformly distributed, and confined to the cavities of the MOF.

## 1. Introduction

The increasing release of carbon dioxide (CO_2_) is a matter of global environmental concern, with intensive efforts aimed at recovering a global carbon balance by using energy generated from renewable sources in preference to traditional fossil fuels. Today, transport consumes most of the world’s petroleum. Although electric vehicles are becoming standard, many regions described as the Global South depend on traditional fuels, with increasing use of biofuels (biodiesel and bioethanol) as alternatives to petroleum. Unfortunately, biodiesel production results in the formation of glycerol (GL) as a by-product of transesterification, with at least 9 kg of GL accumulating per 100 kg of biodiesel produced [1]. The scale of GL production exceeds any reasonable demand: the chemical and pharmaceutical industries utilize less than 20% of the generated glycerol, and the rest is not even purified because the costs of purification exceed the price of the final pure product. The problem of environmental significance and other challenges connected with excess bioglycerol and market dynamics have been highlighted and discussed in several reports and reviews [1,2,3,4]. The most sustainable method for glycerol utilization is to capture CO_2_ by GL and convert both wastes into added-value products. Glycerol carbonate (GLC) appears to be the most versatile valuable product from GL [2,4,5], with a high dielectric constant (111.5), low vapor pressure (0.008 bar at 177 °C), low melting point (−69 °C), low flammability/high flash point (190 °C), negligible toxicity, and high miscibility, among other features.

**Scheme 1:**
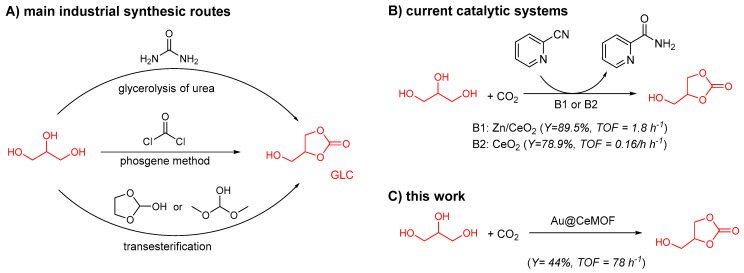
Synthetic pathways to GLC. The TOFs in (**B**) are calculated based on reported data [16,17].

GLC is a clean substitute for alkyl carbonates, and this green solvent [6] has many applications (as a solvent for electrolytes in batteries; for lubricants and plasticizers; for the production of polycarbonates and polyurethanes, membranes, coatings, paints, and surfactants). On an industrial scale, GLC is produced from toxic (and expensive) phosgene (see Scheme 1A). The other two technologies presented in Scheme 1A are also of limited applicability. Transesterification is expensive due to the high cost of organic carbonates used [7,8]. The glycerolysis of urea could be competitive but is not sufficiently sustainable because the industrial production of urea relies on the combustion of large amounts of methane to obtain ammonia. The reaction is shifted to the products by removing generated ammonia under reduced pressure. Therefore, the direct catalytic reaction of GL with CO_2_ to form GLC (Scheme 1B) appears to be the greenest option and has attracted the attention of many research groups [4,9,10,11]. The efforts focused on applying metal and metal-oxide-based catalysts have resulted in relatively small progress [12,13,14,15] as the proposed catalytic materials exhibited TOF parameters are far from satisfactory (see Scheme 1B).

As presented in Scheme 1B, methods B1 and B2 [16,17] are thermodynamically uphill reactions driven forward by the irreversible loss of water due to the use of a stoichiometric excess of expensive 2-cyanopyridine as a dehydrating agent. In both methods, the catalysts are recovered by calcination, and such thermal treatment (at 600 and 400 °C, respectively) increases the overall costs of the process. Furthermore, a hazardous by-product (pyridine-2-carboxamide) is formed that is not readily biodegradable, has a low flash point (89 °C), and during calcination decomposes, causing the release of CO, CO_2_ and nitrogen oxides (NO_x_). The limited attractiveness of these methods caused attention to turn to other kinds of catalytic systems. A different approach to tackling the problem of low catalytic efficiency includes the application of metal nanoparticles (MNPs) combined with metal–organic frameworks (MOFs) and the use of an inert dehydrating agent in order to remove water and avoid the formation of other products, for example, by replacing toxic and expensive 2-cyanopirydine with MgCO_3_. When water is removed physically by MgCO_3_, no toxic by-product is formed and the dehydrating agent might be recovered together with the catalyst, activated by drying, and reused. Surprisingly, this direction is not represented in the rather broad literature on GL/CO_2_ chemistry. MNP-based catalysts are commonly used in both organic and inorganic transformations [18], whereas MOFs are versatile materials in many fields, including catalysis; the sorption of gases [19,20], with catalytic reactions of CO_2_ with olefins [21] or epoxides (obtaining up to 96% yield of propylene carbonate) [22], and the direct cyclodimerization of epichlorohydrin to produce 1,4-dioxane 2,5-bis-chloromethyl [23]. The catalytic applicability of MOFs is usually limited because of their moderate stability and low number of catalytic centers. However, the first weakness can be overcome by improving the architecture of MOFs aimed to increase in chemical and thermal stability (which are usually compromised), and the second drawback can be solved by enriching MOF with more active centers, like MNPs as guests. On the other hand, the idea of combining of MOFs with MNPs can also improve the catalytic activity of MNPs as a convenient protection against agglomeration, oxidation, leaching from the support material [24,25], or poor accessibility to active centers when MNPs are coated with stabilizing surfactants or ligands [26,27,28]. The accessibility/contact of MNPs with reactants could be enhanced by embedding MNPs within the cavities of an MOF [28,29], and such a combination might be facilitated by a proper design of the MOF architecture or its post-synthetic modifications.

Here, we used Cu, Ag, and Au NPs stabilized by a cerium-mixed carboxylate MOF as catalysts for the reaction of glycerol with carbon dioxide to form glycerol carbonate. AuNPs, with their high chemical and physical stability, well-defined morphology [30], and high catalytic activity in numerous transformations, including oxidative carboxylation [31], are frequently used as model NPs to develop new catalytic systems to be further extended on cheaper and more available metals or bimetallic catalysts. Cerium-based catalysts demonstrate exceptional abilities in activating small molecules, including CO_2_ [16,32,33]. Moreover, Ce-based MOFs are inexpensive and more resistant to the presence of oxygen and moisture.

## 2. Materials and Methods

### 2.1. Chemicals and Instrumentation

Detailed information on the chemicals used, their origins, and detailed analytical protocols are given in the Supplementary Materials. UV–vis spectra were recorded using a Thermo Scientific Genesis 50 UV/Vis spectrophotometer (Waltham, MA, USA) in the 200–800 nm range using glass quartz cells (10 mm path length). Infrared spectra (in KBr) were obtained on a Shimadzu FTIR-8400S spectrophotometer (Kyoto, Japan). Using Cu radiation, the crystal structures were characterized using powder X-ray diffraction on a Bruker AXS D8 X-ray diffractometer (Billerica, MA, USA). The diffraction patterns were processed in the TOPAS application and compared with the literature data about MOF-808(Ce), UiO-66(Ce), and CeBTC. N_2_ sorption isotherms were analyzed at 77 K with Autosorb iQ Station 1 (Quantachrome Instruments, Boynton Beach, FL, USA). Before analysis, the samples were degassed at 120 °C for 12 h. The surface area and pore properties were obtained using BET and BHJ methods, respectively. X-ray photo-electron spectroscopy analysis (XPS) was performed at 150 W. High-resolution spectra were obtained using a scan step of 0.2 eV with a pass energy of 200 eV for Au 4f and Ce 3d. The neutralizator was set at 1 eV/1 μA. The catalyst (fine powder) was placed on a conductive carbon tape. The beam was monochromated using an Al Kα (1486.7 eV) monocromator. Curve fitting of the XPS data was carried out using the casaXPS program (version 2.3.15) and Shirley background. For Ce 3d spectrum E Tougard background was used because the energy interval was too weak for reliable Shirley background use.

Scanning electron microscopy (SEM) images and energy-dispersive X-ray spectroscopy (EDS) measurements (SEM-EDS) were performed using three microscopes: a scanning electron microscope JEOL-JSM-5600 (Tokyo, Japan) equipped with an energy-dispersive X-ray spectrometer OXFORD Link-ISIS (Oxford, UK); a Zeiss Merlin FE-SEM scanning electron microscope (Oberkochen, Germany) equipped with a Bruker Quantax XFlash EDS detector 5010 (Billerica, MA, USA) operated at 3 kV and 30 pA for imaging and 15 kV and 500 µA for EDS analysis; and a Jeol JSM—6380LA SEM (Tokyo, Japan) operated at 20 kV with BSE detector, W.D = 16 mm, an aperture size of 120 µm, and a Bruker XFlash 6110 (Berlin, Germany) as the EDS probe. Transmission Electron Microscopy (TEM) images were taken on a Zeiss Libra 120 Microscope (Oberkochen, Germany) operated at 120 kV and on a JEOL 1400 electron microscope (Tokyo, Japan). Inductively coupled plasma-mass spectrometry data were acquired on digested samples. For 12 h, 20 mg of the catalyst was aged at room temperature in 5 mL of acid mixture (0.30 mL of conc HCl in 2 mL 65% HNO_3_). Then the resultant mixture was digested at 180 °C with 2 mL of the acid mixture every 30 min until the solution became transparent and colorless (3 h) and came to a total of 25 mL with the HNO_3_. Samples for analysis were further diluted with 3% HNO_3_. Metal contents were determined on a NexION 300D spectrometer (Perkin Elmer, Waltham, MA, USA) equipped with a liquid sample introduction system. The ICP-MS apparatus was calibrated by measuring a series of reference solutions with concentrations ranging from 10 µgL^−1^ to 200 µgL^−1^ (obtained by diluting ICP standard solutions). Dilution was made using ultra-pure water. The measurement was obtained as a count of ions with a mass-to-charge ratio of appropriate isotopes. The apparatus was purged with a neutral solution for 60 s after each sample and was allowed to equilibrate for 40 s before measurement. Three measurements were obtained for each sample and averaged. The limit of quantification was calculated from the results obtained for the digested blank solutions. The reaction products were analyzed by gas chromatography (GC) using an Agilent 7820A (Santa Clara, CA, USA) chromatograph equipped with an HP-5 capillary column (30 m long × 0.32 mm internal diameter × 0.25 mm filling) and a flame ionization detector (FID).

### 2.2. Synthesis of Nanoparticles Au@PEI and Au@DBU

The preparation of the nanoparticles was based on the synthesis of gold nanoclusters in aqueous solutions using branched polyethylenimine (PEI) as proposed by Tao et al. [34] with further modification: briefly, a solution containing HAuCl_4_·3H_2_O (0.08 g, 0.2 mmol) and PEI (2 g) in ultrapure water (60 mL) was adjusted to about 7 °C in an ice bath. Then, a cold aqueous solution of freshly prepared sodium borohydride (2 mmol) was added with constant stirring (1200 rpm), and the mixture was allowed to react for 1 h. The resultant solution was reddish brown, indicating the formation of AuNPs designated as Au@PEI NPs. In a separate synthesis, Au@DBU NPs were synthesized using 1,8-diazabicyclo [5.4.0]undec-7-ene (DBU) (2 mL, 13 mmol) as a stabilizing ligand instead of PEI. The nanoparticles were precipitated with acetone after a reduction in the solvent volume under reduced pressure, then isolated by centrifugation (15,000 rpm, 20 min), dried, and stored for further use. For TEM measurements, a drop of NP solution in water or ethanol was placed on a carbon-coated copper grid and allowed to dry under ambient conditions before TEM measurements were conducted.

### 2.3. Pre-Synthesized MOF

The MOF was constructed from two organic components, terephthalic acid (BDC) and 1,3,5-benzenetricarboxylic acid (BTC), using the hydrothermal/solvothermal method [35]. BTC (11.2 mg, 0.05 mmol) and BDC (5.8 mg, 0.1 mmol) were ultrasonically dissolved in 3 mL of DMF and mixed with a solution of ammonium cerium (IV) nitrate (13.7 mg, 0.25 mmol) in ultra-pure water (1 mL) under stirring for 5 min. Then formic acid (250 µL) was added under constant mixing. The mixture was sealed in a Teflon-lined steel autoclave, heated to 70 °C, and kept at this temperature for 20 min. The mixture was allowed to cool down and the precipitated MOF was separated by centrifugation, washed twice with DMF and two times with methanol, and afterwards soaked in methanol for 12 h, with the methanol portions being replaced after 6 h. Finally, it was washed once with acetone and allowed to dry in the air (fume-hood) for 1 h. The crystalline powder was dried at 80 °C in a vacuum for 12 h and thermally activated (150 °C for 12 h) before use and characterization. The synthesis of MOF was scaled up with 10-fold higher amounts of reactants.

### 2.4. Synthesis of Catalysts

Three synthetic strategies were applied to combine the MOF and NPs into one catalytic system. **Method 1** is based on the generation of NPs inside the already pre-synthesized MOF. The catalyst obtained by this method is denoted as NP-MOF1, where NP is a nanoparticle of nanoclusters of Au, Ag, and Cu. **Method 2** involves the buildup of MOF around the already pre-synthesized MNPs, and these catalysts are abbreviated as NP-MOF2. The third strategy (**Method 3**) is a one-pot in situ formation of a catalyst from precursors of both MNPs and MOF; such catalysts are described as NP-MOF3.

**Method 1.**  Synthesis of catalysts using pre-synthesized MOF

The NPs or nanoclusters (NCs) of Au@DBU and Au@PEI were embedded in the MOF using simple precursor impregnation method followed by reduction [36]. Pre-synthesized MOF material (75 mg) was ultrasonically dispersed in methanol (15 mL). Measures of 6 mg (0.015 mmol) of HAuCl_4_∙3H_2_O and 100 µL DBU were dissolved in 1 mL of water and added, and the mixture was stirred for 6 h and then placed in an ice bath at about 7 °C before a cold aqueous solution of NaBH_4_ (0.15 mmol, 2 mL) was added. After 30 min of stirring, the resulting solid product was removed by centrifugation at 8000 rpm for 10 min, washed twice with water and once with methanol, dried for 1 h at RT, and then dried overnight at 80 °C to produce approximately 72.5 mg of powder, which was finally reduced/activated in a flow of H_2_/He (1:2 *v*/*v*) at 210 °C (quartz tube) in a horizontal furnace for 1 h before use and characterization. This catalyst is denoted as 4%Au@DBU-MOF1 and contains 4% of Au (calculated with respect to Ce in (NH_4_)_2_Ce(NO_3_)_6_ used, see the analysis of the catalysts in the Results and Discussion). By changing the amount of HAuCl_4_ to 4 mg and 12 mg, two other catalytic systems were obtained: 3%Au@DBU-MOF1) and 8%Au@DBU-MOF1, respectively. The catalyst based on polyethylenimine (4%Au@PEI-MOF1) was synthesized following the same procedure except that 200 mg of PEI was used instead of DBU. Catalysts with silver and copper nanoparticles (2%Ag@PEI-MOF1 and 2%Cu@PEI-MOF1) were synthesized using an appropriate amount of AgNO_3_ or CuSO_4_ instead of Au precursor.

A variation of this procedure, but without surfactants and with gold reduced directly with H_2_ (instead of NaBH_4_), was used for the preparation of the catalyst Au(H_2_)@MOF1, following the double solvent method [37]: 250 µL of aqueous solution of HAuCl_4_∙3H_2_O (6 mg, 0.01 mmol) was added dropwise to ultrasonically dispersed pre-synthesized MOF (85 mg) in 15 mL of dry n-hexane. The reaction vessel was removed from the sonic bath and the mixture was stirred until all the solid was deposed on the walls of the flask to leave colorless n-hexane, which was allowed to stand for 3 h. The solvent was gently removed, and the MOF impregnated with HAuCl_4_ was vacuum dried under ambient conditions, then dried at 70 °C under vacuum for 12 h. Finally, the Au^3+^ impregnated in the MOF was reduced with mixed H_2_/He (approximately 1: 1 *v*/*v*) at 210 °C for 2 h to obtain the final catalysts with approximately 3.4 mol% Au (ICP-MS) based on Ce, which was labeled 4%Au(H_2_)@MOF1.

**Method 2.**  Synthesis of 4%Au@DBU-MOF2

A solution of sodium borohydride (0.06 g, 1.5 mmol) in DMF (5 mL) was added dropwise to a solution containing HAuCl_4_·3H_2_O (0.06 g, 0.15 mmol) and 1,8-diazabicyclo [5.4.0]undec-7-ene (DBU) (1 mL, 6.55 mmol) in DMF (20 mL), immersed in an ice bath. The reaction mixture was stirred and allowed to react for 1 h. The resultant mixture was red, which indicated the formation of AuNPs. The mixture was dispersed in a solution containing 1, 3, 5-benzenetricarboxylic acid (BTC) (0.263 g, 1 mmol) and terephthalic acid (BDC) (0.166 g, 1.2 mmol) in DMF (30 mL) and ammonium cerium(IV) nitrate (3.5 mmol, 10 mL H_2_O) under ultra-sonication and then allowed to react at 120 °C for 1 h. The solid product was recovered by centrifugation and washed with DMF and then with methanol. Afterward, the product (4%Au@DBU-MOF2) was dried at 80 °C in a vacuum oven for 12 h and activated at 150 °C with H_2_.

**Method 3.**  One-pot synthesis of the catalyst

A solution containing HAuCl_4_∙3H_2_O (0.06 g, 0.15 mmol) and 1,8-diazabicyclo [5.4.0]undec-7-ene (DBU) (1 mL, 6.55 mmol) in DMF (20 mL) was stirred for 5 min, and the resultant solution was mixed with a solution containing 0.263 g (1 mmol) of 1, 3, 5-benzenetricarboxylic acid (BTC) and 0.166 g (1.2 mmol) of terephthalic acid (BDC) in 30 mL of DMF and 6 mL (3.5 mmol) aqueous solution of ammonium cerium (IV) nitrate. This was then sonicated, sealed, and allowed to react at 120 °C for 12 h. The solid was recovered by centrifugation and washed with DMF and then with methanol. Afterward, the product was dried overnight at 80 °C in a vacuum. The dried catalyst was activated in H_2_ gas at 150 °C for 1 h. The final material was marked as 4%Au@DBU-MOF3.

### 2.5. Carboxylation of Glycerol with Carbon Dioxide Using the Catalysts

Measures of 10 mL of DMF, 1.15 g (12.5 mmol) of GL, 2.5 mL of methanol, and the dehydrating agent (1.0 g of MgCO_3_, or 2 mL of adiponitrile, or 2.5 mL of acetonitrile) were placed in a stainless-steel autoclave equipped with a Teflon vessel and a magnetic stirring bar. For crude glycerol, 3 mL of the crude GL were diluted to 10 mL with DMF without the addition of methanol or the dehydrating agent (1.0 g of MgCO_3_ was added, see Supplementary Material for chemicals and reagents for information on how crude GL was obtained). The catalyst (0.01, 0.05, 0.10, or 0.15 g) was loaded, and the vessel was purged with CO_2_, pressurized with CO_2_, and placed in a preheated oil bath on a magnetic stirrer. After the required time, the reactor was cooled down and carefully depressurized. The solids were separated from the reaction mixture by centrifugation at 4000 rpm for 3 min and the supernatant was derivatized (silylated) and analyzed by gas chromatography. Derivatization was carried out as follows: 0.50 g of the supernatant from the carboxylation reaction was shaken with approximately 2 g of the silylating reagent (a mixture of pyridine, hexamethyldisilazane, and trimethylchlorosilane at molar ratio of 9:3:1, respectively) in a vial. After 0.5 h, the formed precipitate was separated by centrifugation at 4000 rpm for 2 min, and the supernatant was analyzed by gas chromatography. The conversion of GL and yield of GLC were determined from the integrated peaks for GL and GLC on chromatograms, calculated with respect to the initial amount of GL using the internal standard method and expressed in %. See Supplementary Materials for details on the calculation of GLC yield, GL conversion, TON, and TOF (Equations (S1)–(S6)). Please note that TON/TOF parameters were calculated based on moles of Au embedded in MOF.

## 3. Results and Discussion

### 3.1. Synthesis and Characterization of the Nanoparticles

**Figure 1:**
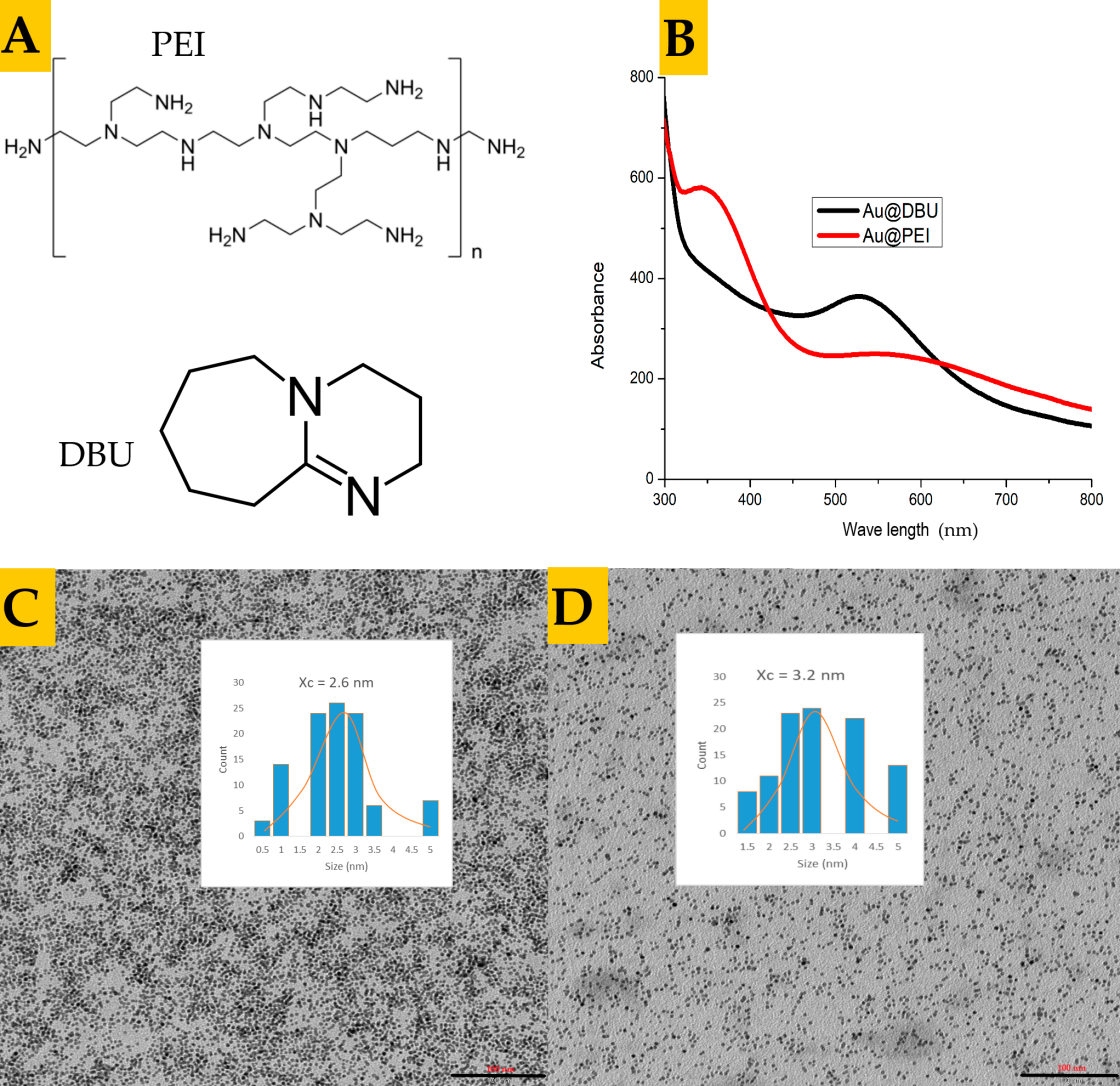
(**A**) Structures of 1,8-diazabicyclo [5.4.0]undec-7-ene (DBU) and polyethylenimine (PEI). (**B**) UV–Vis of the NPs at concentration 1.3 mM (calculated with respect to Au) in water. Lower panels: TEM images with histograms for AuNPs stabilized with PEI (**C**) and DBU (**D**).

Good stabilization and protection of NPs against agglomeration in water was obtained due to the strong interaction with nitrogen-containing ligands PEI [34] and DBU (see Figure 1). The UV–vis absorption spectra (Figure 1B) recorded for Au@DBU indicate a clearly visible localized surface plasmon resonance (LSPR) band at around 530 nm, which suggests that the average size of formed AuNPs is greater than 2 nm. On the other hand, a weak LSPR at 530 nm for Au@PEI indicates the presence of few large nanoparticles, while a strong band with a maximum at ca. 365 nm can be assigned to Au nanoclusters [38,39].

These observations were supported by the TEM measurements presented in Figure 1. AuNPs are spherical and have a very narrow size distribution, with a mean diameter of 3.2 nm for Au@DBU and 2.6 nm for Au@PEI (Figure 1C and Figure 1D, respectively).

### 3.2. Analysis of Pre-Synthesized MOFs

**Figure 2:**
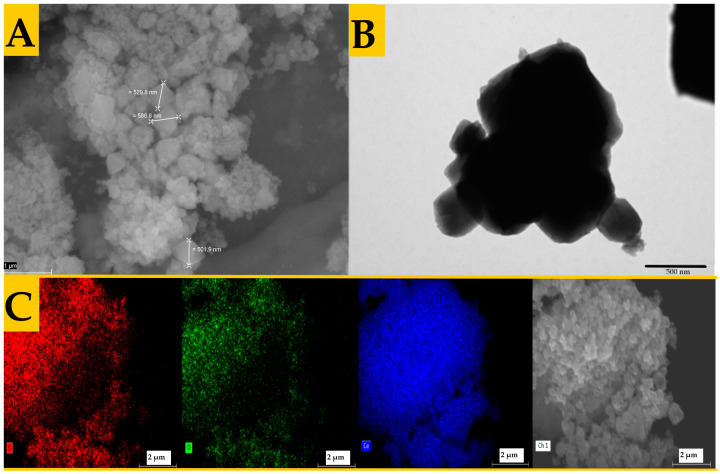
SEM (**A**), TEM (**B**), and SEM-EDS (**C**) elemental maps of O, C, and Ce, respectively, and far right is the image of the prepared Ce-MOF containing BTC and BDC.

Among the rare earth metals, cerium and its compounds are the most accessible, with costs comparable to those of metals like copper. Ce- and CeO_2_-containing catalysts have exceptional abilities to activate small molecules such as CO_2_ [40]; therefore, we decided to prepare cerium-containing MOFs as part of the catalytic system. Chemically and thermally stable MOF-808(Ce) and UiO-66(Ce) and their Zr predecessors can be produced with the BDC and BTC linkers [41], respectively, and we applied this convenient, non-expensive, and environmentally friendly method to prepare Ce-MOF based on both organic components assembled within one framework (see Section 2—Materials and Methods). The SEM, TEM, and XRD images of the synthesized Ce-MOFs are given in Figure 2.

**Figure 3:**
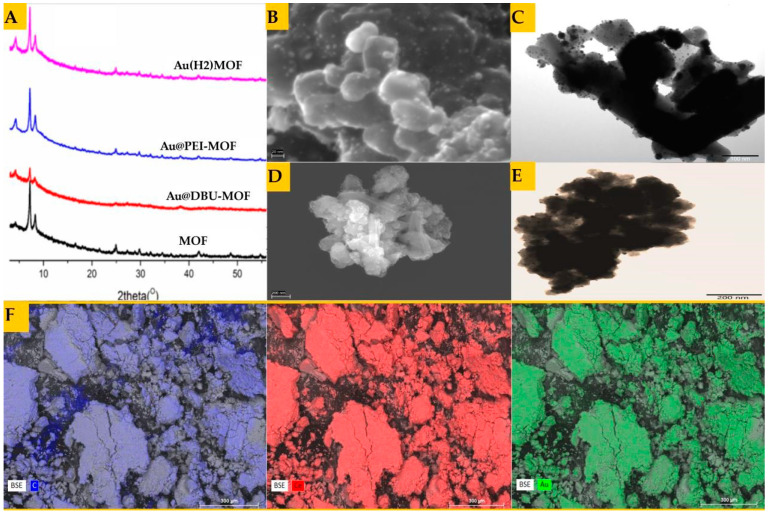
(**A**) XRD powder patterns for the catalysts prepared by method 1 (the lowermost plot is for the pre-synthesized MOF). Panels (**B**–**E**): SEM and TEM images of 4%Au@DBU-MOF1 (**B** and **C**, respectively) and 4%Au@PEI-MOF1 (**D** and **E**, respectively). Panel (**F**): SEM EDS elemental maps of 4%Au(H_2_)-MOF1.

The pure MOF is shown in Figure 2A,B (SEM and TEM, respectively). We expected to obtain a material with a new crystal-like morphology as a result of cerium bridges formed between dicarboxylate and tricarboxylate anions. However, the morphology and size of the synthesized powder particles indicate a mixture of MOF-808(Ce) and UiO-66(Ce) [42], suggesting that two-valent (BDC) and trivalent (BTC) anions do not assemble in one hybrid system, but each of them forms a separate MOF with cerium cations. The TEM image is typical for UiO-66 and MOF-808 with aggregates, and the sizes of the pristine MOFs range from 40 nm to ca. 560 nm (note that the small MOF NPs appear as aggregates in the bigger picture, see Figure S1). The MOF presented in Figure 2A contains a larger number of aggregated crystals that cover the regular octahedral crystals, which is common with UiO-66(Ce), as recently reported [43,44]. Based on this similarity, we suggest that our product contains more UiO-66(Ce) MOF than MOF-808(Ce). This is supported by the comparison of powder X-ray diffraction patterns for mixed MOF and for the two catalysts 4%Ag@PEI-MOF1 and 4%Au(H_2_)@MOF1 presented in Figure 3E, whose XRD results are similar to the diffraction pattern obtained for UiO-66(Ce) [45,46,47].

### 3.3. Synthesis and Characterization of the Catalysts

The aim of all three procedures described in the Section 2—Materials and Methods (Methods 1–3) was to produce catalysts in the form of nanoparticles embedded inside the cavities of MOFs because such a NPs@MOF system ensures the stabilization of the NPs. The catalysts obtained using Method 1 (with pre-synthesized MOFs and abbreviated MOF1) were the most active and selective for the carboxylation reaction, and our discussion will concentrate much on the ones with gold nanoparticles embedded inside cavities: 4%Au@DBU-MOF1, 4%Au@PEI-MOF1, and 4%Au(H_2_)@MOF1. The FTIR spectra (shown in Figure S2) indicate strong bands at 1401, 1596 cm^–1^ and 1421, 1576 cm^−1^ in all the samples due to symmetric and antisymmetric carboxylate stretching modes [48,49]. The appearance of weak bands at around 3000 to 3500 cm^−1^ in all spectra is attributed to both O-H (from adsorbed water) and C-H stretching modes or N-H bonds present in 4%Au@DBU-MOF1 and 4%Au@PEI-MOF1. The weak bands at 524, 750, and 110 cm^−1^ are due to the several vibrational modes of the benzene rings from the carboxylate linkers in all of the spectra.

As presented in Figure 3A, the XRD patterns demonstrate similarity regarding their XRD peaks and their 2theta positions to UiO-66(Ce) [45,46,47], UiO-66(Hf) [50], and MOF-808(Ce) [51] (see Figure S3 in Supplementary Materials), confirming that our catalysts were obtained with good purity. Apart from the XRD pattern of 4%Au@DBU-MOF1 (Figure 1A), the rest of the plot represents sharp peaks and therefore high crystallinity.

As can be seen in Figure 3A, there is no appreciable difference between the XRD results of the MOF and the three catalysts based on this MOF: 4%Au@DBU-MOF1, 4%Au@PEI-MOF1, and 4%Au(H_2_)-MOF1. Such retention of the XRD pattern of the MOF after the introduction of Au [50,52] corroborates our expectations that AuNPs are present at a low concentration (confirmed by ICP-MS), uniformly distributed, and held inside the MOF, resulting in no X-ray reflections from Au. The XRD pattern of 4%Au@DBU-MOF1 suggests that the material is amorphous, probably due to the interference of reflection from the much higher number of AuNPs on the surface of the MOF, as is evident in the TEM and SEM images (Figure 3B,C). Large NPs (about 5 to 10 nm) are visible on the catalyst’s surface, while small ones are inside the MOF. No small AuNPs are visible on the surface, suggesting they were effectively embedded inside the MOF cavities. Obstruction due to overlap and masking by the host MOF surface could not allow for the concise particle positioning of very small NPs/NCs using TEM imaging [53,54,55].

Large NPs reside tightly on or close to the surface of the MOF crystals, probably due to an ineffective encapsulation strategy that might have resulted in some Au NPs on the surface of the MOF aggregating during synthesis or TEM imaging [53,54,55]. In contrast, the XRD of the 4%Au@PEI-MOF1 shows sharp peaks (Figure 3A), pointing to better crystallinity than the DBU catalysts, and is complemented by the TEM images of the PEI catalyst, which shows no particles on the surface. In some images (see Figure 3B–D), we observed the presence of a few rod-like crystals and needle-like forms similar to the BTC-based Ce-MOF reported by other researchers [56], suggesting a presence of Ce^3+^ moieties during synthesis [56]. The 4%Au@PEI-MOF1 and 4%Au(H_2_)@MOF1 form more regular crystals, and there is a smaller number of aggregates than in the pristine MOF, suggesting that the presence of the NPs prevents aggregation in the MOFs.

The SEM image of the 4%Au(H_2_)@MOF1 synthesized by a modified double solvent method coupled with the hydrogen reduction of Au^3+^ is given in Figure S4 (Supplementary Materials). No AuNPs are present on the surface of the MOF crystals. This shows that this strategy is more efficient in embedding the ultra-small NPs inside the MOF. Moreover, the crystals of the MOF seem to maintain a uniform shape. This probably explains the better performance and reusability of the resultant catalyst, vide infra. The EDS elemental maps shown in Figure 3F support the speculation of the presence of NCs. The EDS elemental maps of the MOF 4%Au(H_2_)@MOF1 given in Figure S5 confirm that Ce and Au are uniformly distributed in the catalysts. This suggests that all the Au is well distributed inside the MOF. The representative elemental composition is given in the Supplementary Materials (Figure S6).

**Figure 4:**
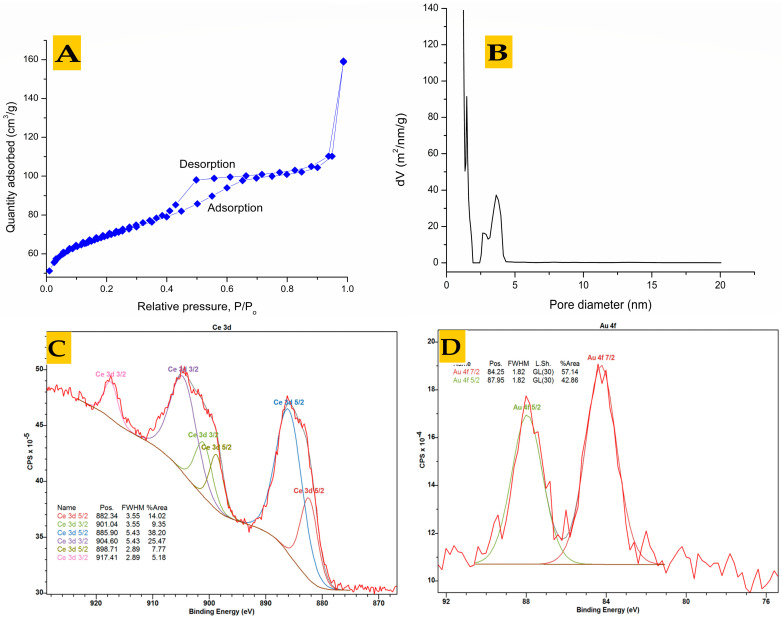
Physisorption analysis of the MOF: (**A**) N_2_ adsorption/desorption plot and (**B**) pore size distribution;. Lower panels: XPS spectra of Au(H_2_)@MOF1 for: (**C**) Ce and (**D**) Au (with Shirley background subtracted).

Measurements of the MOF’s porosity and surface area and a pore analysis were also conducted. As presented in Figure 4A, the N_2_ adsorption–desorption graph and the pore size distribution graph provide an estimated total surface area of the MOF of 459 m^2^/g, and a pore volume of 0.156 cm^3^/g. The pore size distribution shows both micropores and mesopores due to the presence of volume in both regions with more mesopores, as indicated by the distribution plot (approximately 0.5 to 20 nm with two peaks at around 1 nm and 3.5 nm pore size) (Figure 4B). The adsorption plot shows a type 4 isotherm with an H_2_a hysteresis loop at a relative pressure of approximately 0.37–0.7, according to the latest IUPAC classification [57]. This type of hysteresis loop is caused by the blocking of the desorption pores and delayed condensation during the physisorption measurement due to connectivity in the pores with small apertures/necks [57].

The XPS spectra of Au 4f were taken for the most promising system: Au(H_2_)@MOF1. NP–support interactions usually play a very important role in supported/composite catalysts [58,59,60,61]. Therefore, XPS analysis was performed mainly to determine the chemical states of Au and Ce atoms and the relative percentage composition in the composite catalyst because it is a reliable technique for studying the surface chemistry of the elements. Such information is relevant to checking the presence of the species in reduced or oxidized states and the presence of NP–support interactions. The XPS spectra of Ce and Au are given in Figure 4C and Figure 4D, respectively, while the survey spectra are given in the Supplementary Materials (Figure S8). From the survey spectra of the sample, peaks confirming the presence of the major elements, i.e., for Au 4f, Ce 3d, C 1s, and O 1s, are evident. The relative quantities in at% are C = 66.4, O = 24, Au = 0.15, and Ce = 2.05. The high-resolution XPS spectra in Figure 4D show binding energies of 87.95 eV (Au 4f5/2) and 84.25 (Au 4f7/2) for Au^0^. On the other hand, the peak at 885.9 eV in Figure 4C is due to Ce^3+^ [62,63,64] and contributes 38.2% of the area, which is higher than all other Ce states. Presence of Ce^3+^ in Ce^4+^ MOFs and ceria creates structural defects (oxygen vacancies), resulting in higher catalytic activity. The surface defects in the MOF can strongly interact with Au atoms and modify their electronic structure to stabilize the oxidation states of Au [62,63,64], which could play a role in the insertion of CO_2_ into the alcohol. Therefore, we examined the chemical status of Au in the synthesized catalyst composite. As depicted in Figure 4C, the deconvoluted Au 4f spectra exhibit two main peaks corresponding to metallic Au^0^. No dominant peaks for Au^3+^ (normally at around 86.1 and 90.2 eV) are observed, confirming the presence of most of the Au in the zero state [65]. However, there are minor peak branches on the main peaks of the Au binding energy, which are obviously assigned to oxidized Au (Au^3+^ and Au^+^). Since it is very difficult to have oxidized Au under an excess of H_2_ and at 205 °C, the presence of oxidized gold species in the XPS spectra must originate from the strong Au–CeMOF interactions caused by the defects [66,67,68]. Moreover, the NPs not surrounded by agents containing heteroatoms have better contact with Ce-O moieties in the MOF. Similar effects were observed for Au supported on ceria [68]. Gold existing at different oxidiation states in AuNPs [69] accelerates the charge transfers leading to superior catalytic performance, and were also reported to enhance CO_2_ adsorption [63,66]. It is also important to note that the characteristic peaks for C and Ce (Figure S8) of the MOF are not attenuated, which confirms the efficient encapsulation of Au NPs inside the CeMOF, not on the MOF surface.

### 3.4. Catalytic Activity of Synthesized X%AuNPs@MOF

The direct carboxylation of GL with CO_2_ is known to form various products depending on the reaction conditions. In this work, effort was put into optimizing the conditions towards selective GLC formation, though the identification, isolation, and quantification of other products is outside the scope of this study, as the by-products of glycerol carbonylation (acetins, dimethyl carbonate, and glyceraldehyde) have been well identified and described in several reviews [4,10,16]. Since the analytical standards of GL and GLC are commercially available, we were able to identify and quantify these compounds accurately by gas chromatography using the internal standard method.

Optimization experiments of reaction conditions for the carbonylation of GL to GLC with carbon dioxide in the presence of X%Au@DBU-MOF1 (X = mol% of Au, see Section 2—Materials and Methods) included the determination of the effect of various factors (dehydrating agent, catalyst composition and amount, CO_2_ pressure, temperature, time) on the conversion of GL and the yield of GLC.

#### 3.4.1. Effect of a Dehydrating Agent, Au Content, and Amount of Catalyst

Dehydration is crucial for the carbonylation of GL to GLC by CO_2_ [3,70,71] because the evolved water shifts the equilibrium towards reactants. The effect of dehydrating agents (MgCO_3_, acetonitrile, and adiponitrile) on the conversion of GL and the GLC yield was tested, and the results are presented in Table 1. Since mass transfer limitations are more pronounced in reactions involving a mixture of materials in different physical states, we checked the stirring speed and the optimal mass of MgCO_3_ to ensure the absence of such limitations. Using 0.5 g of MgCO_3_ gave a very low conversion, and the highest conversion and uniform stirring was observed when 1 g of MgCO_3_ was used. Increasing the mass to 1.5 g had no effect on stirring uniformity and no effect on conversion, whereas the presence of 2 g of MgCO_3_ caused non-uniform stirring, sluggish and depositing lumps on higher parts of the vessel were observed. Thus, 1 g was chosen as an optimal amount of MgCO_3_ for further experiments.

The highest yield was obtained for MgCO_3_ (perhaps due to the stability of MgCO_3_ in the protic environment [72]), followed by adiponitrile. We observe a much better performance for adiponitrile than for acetonitrile, and such results agree with the data in the literature [73]. Considering the results from Table 1, we decided to study the catalytic activity of other catalysts in the presence of the two most effective dehydrating agents (MgCO_3_ and adiponitrile) (see runs 1–5 in Table 2). The added advantage of using MgCO_3_ is that it captures the formed water from the reaction mixture physically without undergoing a reaction to form other products.

Three catalysts based on MOF1, i.e., 4%Au(H_2_)@MOF1, 4%Au@PEI-MOF1, and 4%Au@DBU-MOF1, exhibit relatively similar activity (with 44, 42, and 39% yield, respectively). Considering the slight increase in yield in the presence of methanol (Table 1, run 1 versus run 4), methanol was applied as a co-solvent since it can be recovered easily if required. There is an additional advantage of using methanol as this solvent might also form dimethyl carbonate (DMC) [74]. As we did not detect DMC in the reaction mixture, we suppose that all DCM (if formed) is subsequently trans-esterified into glycerol carbonate. The high selectivity of 4%Au(H_2_)@MOF1 can be attributed to the small and evenly distributed Au particles (clusters or nanoparticles) within the MOF cavities, with relatively uniform particle sizes and shapes. The reaction performed with crude glycerol (as a substrate) gave a relatively good conversion (C_GL_ = 49%) but a very poor yield (Y_GLC_ = 7.0%) of GLC (Table 2, run 13). A representative GC chromatogram showing the peaks of the silylated products is given in the Supplementary Materials (Figure S7). The TEM images of the 4%Au@DBU-MOF1 and 4%Au@PEI-MOF1 catalysts indicate the presence of NPs within the MOF (Figure 3B and Figure 3D, respectively). However, most large NPs are visible on or close to the surface of the MOF (Figure 3B), and their broad size distribution has been reported to lead to by-products [75,76]. For reduced or doubled Au content (3%Au@DBU-MOF1 or 8%Au@DBU-MOF1, respectively), conversions and yields changed to 41 and 68% and 28 and 43%, respectively (Table 2, runs 11 and 12). The content of Au = 4% (based on the results obtained for 4%Au@DBU-MOF1) was optimal, and therefore, the same content of Au was applied for the synthesis of 4%Au(H_2_)@MOF1, 4%Cu@ PEI -MOF1, and 4%Ag@ PEI -MOF1 (runs 5, 7, and 8 respectively).

Table 3 presents the effects of the amount of catalyst 4%Au@DBU-MOF1 on its activity in the carbonylation of GL to GLC. As doubling the catalyst amount from 0.05 to 0.1 causes a small change in conversion and yield for the process carried out for 13 h at 150 °C), the optimal amount of catalyst is 0.05 g.

#### 3.4.2. Effect of the Reaction Time, Temperature, and Pressure

Based on the results in Table 2, we selected 4%Au@DBU-MOF1 as the most promising catalyst (before 4%Au@PEI-MOF1 and 4%Au(H_2_)@MOF1 were synthesized) and checked to what degree the reaction efficiency depends on the reaction time, temperature, and pressure, and the results are presented in Table 4. For processes carried out at 150 °C under 1.5 MPa pressure, an increase in GLC yield from 5 to ca. 40% is observed during the first 13 h, and after that time the further increase is negligible (runs 6–11), probably due to decomposition of GLC or side reactions of GLC, as suggested by gas chromatography analysis (small unidentified peaks appear in the products obtained for a long reaction time). Table 4 also presents the results obtained at different temperatures—from 120 to 170 °C (runs 12 to 16), indicating an increase in conversion and yield with an increase in temperature in the range of 120 to 150 °C (runs 14 to 16). However, above 150 °C, the yield of GLC decreases (runs 12 and 13), perhaps due to the formation of by-products formed during the decomposition of GLC or the reaction of GLC with GL at higher temperatures (the conversion of GC still increases). The effect of the third parameter, the pressure of CO_2_ (in the range of 0.5 MPa to 5.0 MPa, see runs 1 to 5 in Table 4), allows one to identify 1.5 MPa as the optimal pressure, as a further increase in CO_2_ pressure causes a high increase in conversion but a negligible increase in yield.

#### 3.4.3. Regeneration and Efficiency of the Applied Catalysts

In order to examine whether the catalysis was heterogeneous or homogeneous (due to a release of nanocatalysts in the form of nanoclusters from the MOF), the leaching from the 4%Au(H_2_)@MOF1 was checked by hot filtration of the reaction mixture at midway through the reaction. To the fresh filtrate (approx. 12 mL), 1.15 g of GL was added and 1 mL of sample was removed for analysis (after homogenization), while the rest was placed in the reactor with fresh MgCO_3_ (1 g), pressurized with CO_2_, and heated to 150 °C over 15 h, and after that time, no conversion of GL was observed, indicating that no appreciable amount of NPs or NCs leached from the MOF to the reaction mixture. This is confirmed by ICP-MS analysis of the fresh and recycled catalysts, which shows negligible difference in the % ratio of Au to Ce after three consecutive cycles. Both experiments indicate also that AuNPs are not released from the MOF, which is an important observation when a decrease in activity is discussed, vide infra.

**Figure 5:**
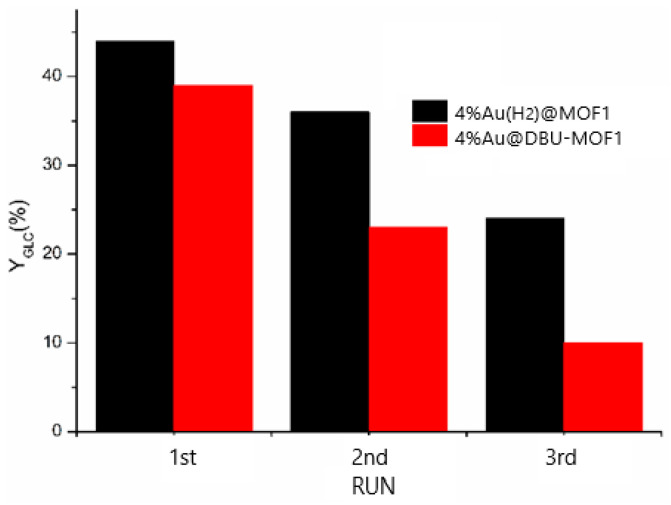
GLC yield over three consecutive cycles in the presence of 4%Au(H_2_)@MOF1 (black bars) and 4%Au@DBU-MOF1 (red bars).

The separation and recyclability of a catalyst from the products have a crucial implication on the profitability and sustainable application of such a catalyst in industry. To recycle the catalyst, the post-reaction solid residue was washed with dry DMF, dried, cleaned in H_2_ as described in the Materials and Methods section, and reused. The activity of 4%Au(H_2_)@MOF1 and 4%Au@DBU-MOF1 (expressed as GLC yield) over three consecutive cycles is shown in Figure 5. Both catalysts lose their activity, but the decrease in yield is smaller for 4%Au(H_2_)@MOF1 (from 44% to 24% after three cycles) compared to the DBU-based catalyst (from 39 to 9%) after the same number of cycles. The better stability of 4%Au(H_2_)@MOF1 is probably attributed to the fine structure of the catalyst, as depicted by the electron microscopy images, which leads to the stability and reusability of the nanocatalysts.

However, the loss of activity observed for the DBU-based catalyst is believed to be due to the loss of nanoparticles from the MOF leading to a decrease in AuNP content during subsequent carboxylation cycles [77]. This is in agreement with the SEM and TEM images (Figure 3B and Figure 3C, respectively), where the particles are seen mainly on the surface of the MOF.

During the last three decades, AuNPs have been used as model catalysts in many reactions, and the results and ideas tested on AuNPs have usually been applied to develop new families of catalysts based on less-expensive metals and their bimetallic combinations. Here, we have demonstrated for the first time that AuNPs@MOF can catalyze the reaction of GL with CO_2_. The main advantage of the catalytic system designed and prepared by us is that a small amount of Au catalyst is required to obtain a reasonable yield, resulting in a TOF that is higher than other catalytic systems reported previously [16,17] by almost two orders of magnitude (>78 h^−1^) (see Scheme 1). Another advantage of the AuNPs@MOF composite is that it requires a low overpressure of 1.5 MPa, compared to 4–8 MPa in other systems [4,10,78], which can be attributed to a synergy of the MOF’s exceptional ability to adsorb CO_2_ and the presence of AuNPs embedded within the MOF. The formation of such unique catalytic centers is fundamentally essential for the future optimization of industrial catalysts. The proposed AuNPs@MOF catalytic system provides a relatively high productivity that is accompanied by a low E-factor, i.e., (mass of waste)/(mass of desired product), because water is the only by-product (the dehydrating agent can be recycled). Taking into account that the other catalytic systems require a long activation time or calcination treatments at elevated temperatures (up to 600 °C), the method of AuNPs @ MOF formation proposed in this work is time and energy saving: preparation in aqueous solution, activation at low temperature, and relatively simple recovery make it a very promising catalytic system that is in line with the assumptions of green chemistry and sustainable processing [79,80]. The catalyst system uses a very dilute amount of Au (0.0064 mmol) and Ce (ca. 0.2 mmol) to yield a high amount of GLC (ca. 50%) based on a calculation made with the use of ICP-MS quantification analysis for 50 mg of catalyst used in the carbonylation of 12.5 mmol of GL.

The catalytic system (Au-based) developed and optimized by us was extended to robust and sustainable systems containing less-expensive metals, i.e., copper and silver (AgNPs and CuNPs). We obtained 25 and 33% GL conversions and 17 and 26% yields for Cu and Ag, respectively. The results are promising given that the reaction system was not optimized for these two metals since the scope of this study was centered on Au.

Higher GLC yields were obtained using other catalytic systems; for example, Lim et al. reported a high GLC yield (86%) using a metal-free catalyst (DBU and CH_2_Br_2_) [81]. They applied very mild conditions (70 °C, 1 MPa); however, CH_2_Br_2_ is expensive and brominated by-products are formed. Another catalytic system based on divinylbenzene 3-butyl-1-vinylimidazolium bromide polymer allows for GLC to be obtained at a yield of 81% [82]; however, the relatively high content of bromide in the polymer (more than 17%), the large amounts of chloroform used, and the complicated and time-consuming synthesis make this method uncompetitive in the current shift to green synthesis.

## 4. Conclusions

In this research, we developed the idea of a catalytic system assembled from two morphologically different solid species, namely metal organic frameworks and nanoparticles, and we tested the applicability of such a system to the carboxylation of glycerol. Several new catalytic systems based on cerium carboxylate MOFs with embedded AuNPs were successfully used to react glycerol with CO_2_ to form glycerol carbonate as the main product. The most efficient were two catalysts prepared from pre-synthesized MOF impregnated with Au^3+^ salt and subsequently reduced to AuNPs using H_2_ (catalyst 4%Au(H_2_)@MOF1) or reduced with NaBH_4_ (catalyst 4%Au@PEI-MOF1). Although the yield of the process is still not competitive when compared to existing systems, the great and most promising advantage is the highest TOF (>78 h^−1^) ever reported for this reaction, which demonstrates the much higher performance compared to already known systems. We also tried to test the applicability of the optimal system to crude bioglycerol because it is more environmentally and economically sustainable but hitherto has been ignored. The conversion obtained is promising, but the system needs optimization to obtain significant GLC yields. Other advantages of such hybrid catalysts include the fast and straightforward fabrication of AuNPs@MOF at a much lower temperature than currently used catalysts and mild conditions of operation (they work at the lowest CO_2_ overpressure ever reported). The developed catalyst system was finally applied to Ag and Cu with promising results (26 and 17% GLC yields, respectively).

For the thermodynamically demanding reaction of CO_2_ with glycerol, the equilibrium has to be shifted toward the products by applying drying agents that drive water out of the reaction mixture, and we found that an inorganic dehydrating agent (MgCO_3_) is more effective than adiponitrile and acetonitrile. The use of the inorganic drying agent brings an additional advantage because, unlike most other methods, no by-product is obtained. The presented preliminary data are promising, and further efforts are planned to ensure the more effective encapsulation of different metal NPs inside MOFs to enable better yields, longer catalyst life, and more-efficient recovery after the reaction.

## Figures and Tables

**Table 1:** The effect of dehydrating agents on the conversion of glycerol (C_GL_) and yield of glycerol carbonate (Y_GLC_) during the carbonylation of GL to GLC *^a^*.

Run	Dehydrating Agent	C_GL_ [%]	Y_GLC_ [%]
1 *^b^*	MgCO_3_	49	32
2	Acetonitrile *^c^*	75	12
3	Adiponitrile *^c^*	63	30
4	MgCO_3_	51	39
5	-	trace	-

*^a^* Reaction conditions: 4%Au@DBU-MOF1, 12.5 mmol GL, 10 mL DMF, 2.5 mL MeOH, 1 g MgCO_3_, 150 °C, 1.5 MPa CO_2_, 13 h. *^b^* Reaction performed in the absence of MeOH, *^c^* 2 mL.

**Table 2:** Conversion of GL (C_GL_) and yield of GLC (Y_GLC_) in the carbonylation of GL to GLC in the presence of CO_2_, MgCO_3_, adiponitrile, and various catalysts *^a^*.

Run	Catalyst	Dehydrating Agent	C_GL_ [%]	Y_GLC_ [%]
1	4%Au@DBU-MOF1	MgCO_3_	51	39
2	4%Au@DBU-MOF1	Adiponitrile *^b^*	63	30
3	4%Au@PEI-MOF1	MgCO_3_	65	42
4	4%Au@PEI-MOF1	Adiponitrile *^b^*	70	37
5	4%Au(H_2_)@MOF1	MgCO_3_	52	44
6	MOF	MgCO_3_	14 *^c^*	12
7	4%Cu@PEI-MOF1	MgCO_3_	25 *^d^*	17
8	4%Ag@ PEI-MOF1	MgCO_3_	33 *^d^*	26
9	4%Au@DBU-MOF2	MgCO_3_	49 *^e^*	40
10	4%Au@DBU-MOF3	MgCO_3_	26	18
11	3%Au@DBU-MOF1	MgCO_3_	41	28
12	8%Au@DBU-MOF1	MgCO_3_	68	43
13 *^f^*	4%Au(H_2_)@MOF1	MgCO_3_	49	7.0
14	-	MgCO_3_	<1	-

*^a^* Reaction conditions: 12.5 mmol GL, 10 mL DMF as a solvent, 2.5 mL MeOH, 1 g MgCO_3_, 150 °C, 1.5 MPa of CO_2_, 13 h. *^b^* 2 mL, *^c^* reaction time = 25 h, *^d^* reaction time = 20 h, *^e^* reaction time = 18 h. *^f^* Instead of pure glycerol, dry crude glycerol was applied (dried over MgCO_3_ and molecular sieves).

**Table 3:** Effect of the amount of catalyst (4%Au@DBU-MOF1) on the conversion of GL (C_GL_) and the yield of GLC (Y_GLC_) *^a^*.

Run	Catalyst Amount [g]	C_GL_ [%]	Y_GLC_ [%]
1	0.01	34	22
2	0.05	51	39
3	0.10	58	41
4	0.15	66	44

*^a^* Reaction conditions: 12.5 mmol GL, 10 mL DMF as solvent, 2.5 mL MeOH, 1 g MgCO_3_, 150 °C, 1.5 MPa CO_2_, 13 h.

**Table 4:** Effect of reaction time (t), temperature (T), and CO_2_ pressure (p) on the conversion of GL (C_GL_) and yield of GLC (Y_GLC_) in the presence of 4%Au@DBU-MOF1 *^a^*.

Run	t [h]	T [°C]	p [MPa]	C_GL_ [%]	Y_GLC_ [%]
1	13	150	5.0	86	38
2	13	150	3.5	76	42
3	13	150	2.5	64	33
4	13	150	1.5	51	39
5	25	150	0.5	39	24
6	18	150	1.5	53	38
7	15	150	1.5	52	40
8	13	150	1.5	51	39
9	9	150	1.5	32	24
10	6	150	1.5	23	16
11	3	150	1.5	13	5
12	13	170	1.5	67	29
13	13	160	1.5	60	34
14	13	150	1.5	51	39
15	13	140	1.5	36	24
16	13	120	1.5	22	7

*^a^* Reaction conditions: 12.5 mmol GL, 10 mL DMF, 2.5 mL MeOH, 1 g MgCO_3_.

## Data Availability

Data are contained within the article and Supplementary Materials.

## Supplementary Materials

The following supporting information can be downloaded at: https://www.mdpi.com/article/10.3390/nano14080650/s1. Description of chemicals; calculation of yield, conversion, TOF, and TON; Table S1: SEM image of 4%Au(H_2_)@MOF1; Figure S1: SEM images showing small MOF nanoparticles of less than 100 nm (A) and large octahedral particles surrounded by aggregates (small particles) (B); Figure S2: FTIR of the synthesized MOF and catalysts materials.; Figure S3: Literature XRD patterns of two MOFs and their derivatives; Figure S4: SEM image of 4%Au(H_2_)@MOF1; Figure S5: SEM-EDS elemental maps for 4%Au(H_2_)@MOF1; Figure S6: EDS elemental composition for 4%Au(H_2_)@MOF1; Figure S7: Gas chromatogram of silylated glycerol and glycerol carbonate; Figure S8: Survey XPS spectra of 4%Au(H_2_)@MOF1. Refs [83,84] are cited in Supplementary Materials.

## References

[1] Monteiro M.R., Kugelmeier C.L., Pinheiro R.S., Batalha M.O., da Silva César A. (2018). Glycerol from biodiesel production: Technological paths for sustainability. Renew. Sustain. Energy Rev..

[2] Sonnati M.O., Amigoni S., de Givenchy E.P.T., Darmanin T., Choulet O., Guittard F. (2013). Glycerol carbonate as a versatile building block for tomorrow: Synthesis, reactivity, properties and applications. Green Chem..

[3] Christy S., Noschese A., Lomelí-Rodriguez M., Greeves N., Lopez-Sanchez J.A. (2018). Recent progress in the synthesis and applications of glycerol carbonate. Curr. Opin. Green Sustain. Chem..

[4] Lukato S., Kasozi G.N., Naziriwo B., Tebandeke E. (2021). Glycerol carbonylation with CO_2_ to form glycerol carbonate: A review of recent developments and challenges. Curr. Res. Green Sustain. Chem..

[5] Tundo P., Musolino M., Aricò F. (2018). The reactions of dimethyl carbonate and its derivatives. Green Chem..

[6] Zhou C.-H.C., Beltramini J.N., Fan Y.-X., Lu G.M. (2008). Chemoselective catalytic conversion of glycerol as a biorenewable source to valuable commodity chemicals. Chem. Soc. Rev..

[7] Ochoa-Gómez J.R., Gómez-Jiménez-Aberasturi O., Maestro-Madurga B., Pesquera-Rodríguez A., Ramírez-López C., Lorenzo-Ibarreta L., Torrecilla-Soria J., Villarán-Velasco M.C. (2009). Synthesis of glycerol carbonate from glycerol and dimethyl carbonate by transesterification: Catalyst screening and reaction optimization. Appl. Catal. A Gen..

[8] Ochoa-Gómez J.R., Gómez-Jiménez-Aberasturi O., Ramirez-Lopez C., Belsué M. (2012). A brief review on industrial alternatives for the manufacturing of glycerol carbonate, a green chemical. Org. Process Res. Dev..

[9] Su X., Lin W., Cheng H., Zhang C., Wang Y., Yu X., Wu Z., Zhao F. (2017). Metal-free catalytic conversion of CO_2_ and glycerol to glycerol carbonate. Green Chem..

[10] Rozulan N., Halim S.A., Razali N., Lam S.S. (2022). A review on direct carboxylation of glycerol waste to glycerol carbonate and its applications. Biomass Convers. Biorefinery.

[11] Procopio D., Di Gioia M.L. (2022). An Overview of the Latest Advances in the Catalytic Synthesis of Glycerol Carbonate. Catalysts.

[12] Aresta M., Dibenedetto A., Nocito F., Pastore C. (2006). A study on the carboxylation of glycerol to glycerol carbonate with carbon dioxide: The role of the catalyst, solvent and reaction conditions. J. Mol. Catal. A Chem..

[13] George J., Patel Y., Pillai S.M., Munshi P. (2009). Methanol assisted selective formation of 1, 2-glycerol carbonate from glycerol and carbon dioxide using *^n^*Bu_2_SnO as a catalyst. J. Mol. Catal. A Chem..

[14] Ozorio L.P., Pianzolli R., da Cruz Machado L., Miranda J.L., Turci C.C., Guerra A.C., Souza-Aguiar E.F., Mota C.J. (2015). Metal-impregnated zeolite Y as efficient catalyst for the direct carbonation of glycerol with CO_2_. Appl. Catal. A Gen..

[15] Ozorio L.P., Mota C.J. (2017). Direct carbonation of glycerol with CO_2_ catalyzed by metal oxides. ChemPhysChem.

[16] Kulal N., Vetrivel R., Ganesh Krishna N.S., Shanbhag G.V. (2021). Zn-Doped CeO_2_ Nanorods for Glycerol Carbonylation with CO_2_. ACS Appl. Nano Mater..

[17] Liu J., Li Y., Zhang J., He D. (2016). Glycerol carbonylation with CO_2_ to glycerol carbonate over CeO_2_ catalyst and the influence of CeO_2_ preparation methods and reaction parameters. Appl. Catal. A Gen..

[18] Narayan N., Meiyazhagan A., Vajtai R. (2019). Metal nanoparticles as green catalysts. Materials.

[19] Dhakshinamoorthy A., Garcia H. (2012). Catalysis by metal nanoparticles embedded on metal–organic frameworks. Chem. Soc. Rev..

[20] Yang Q., Xu Q., Jiang H.-L. (2017). Metal–organic frameworks meet metal nanoparticles: Synergistic effect for enhanced catalysis. Chem. Soc. Rev..

[21] Nguyen H.T., Tran Y., Nguyen H.N., Nguyen T.C., Gándara F., Nguyen P.T. (2018). A series of metal–organic frameworks for selective CO_2_ capture and catalytic oxidative carboxylation of olefins. Inorg. Chem..

[22] Li P.-Z., Wang X.-J., Liu J., Lim J.S., Zou R., Zhao Y. (2016). A Triazole-Containing Metal–Organic Framework as a Highly Effective and Substrate Size-Dependent Catalyst for CO_2_ Conversion. J. Am. Chem. Soc..

[23] Mousavi B., Chaemchuen S., Phatanasri S., Chen C., Zeng C., Ganguly R., Zhuiykov S., Verpoort F. (2020). Selective cyclodimerization of epichlorohydrin to dioxane derivatives over MOFs. Arab. J. Chem..

[24] Farrusseng D., Tuel A. (2016). Perspectives on zeolite-encapsulated metal nanoparticles and their applications in catalysis. New J. Chem..

[25] Ndolomingo M.J., Bingwa N., Meijboom R. (2020). Review of supported metal nanoparticles: Synthesis methodologies, advantages and application as catalysts. J. Mater. Sci..

[26] Cao A., Lu R., Veser G. (2010). Stabilizing metal nanoparticles for heterogeneous catalysis. Phys. Chem. Chem. Phys..

[27] Zhu Q.-L., Xu Q. (2016). Immobilization of ultrafine metal nanoparticles to high-surface-area materials and their catalytic applications. Chem.

[28] Zanon A., Verpoort F. (2017). Metals@ ZIFs: Catalytic applications and size selective catalysis. Coord. Chem. Rev..

[29] Gao C., Lyu F., Yin Y. (2020). Encapsulated metal nanoparticles for catalysis. Chem. Rev..

[30] Lesiak P., Bednarska K., Lewandowski W., Wójcik M., Polakiewicz S., Bagiński M., Osuch T., Markowski K., Orzechowski K., Makowski M. (2019). Self-Organized, One-Dimensional Periodic Structures in a Gold Nanoparticle-Doped Nematic Liquid Crystal Composite. ACS Nano.

[31] Du Y., Sheng H., Astruc D., Zhu M. (2020). Atomically Precise Noble Metal Nanoclusters as Efficient Catalysts: A Bridge between Structure and Properties. Chem. Rev..

[32] Kehoe A.B., Scanlon D.O., Watson G.W. (2011). Role of lattice distortions in the oxygen storage capacity of divalently doped CeO_2_. Chem. Mater..

[33] Misch L.M., Kurzman J.A., Derk A.R., Kim Y.-I., Seshadri R., Metiu H., McFarland E.W., Stucky G.D. (2011). C–H bond activation by Pd-substituted CeO_2_: Substituted ions versus reduced species. Chem. Mater..

[34] Tao Y., Li Z., Ju E., Ren J., Qu X. (2013). Polycations-functionalized water-soluble gold nanoclusters: A potential platform for simultaneous enhanced gene delivery and cell imaging. Nanoscale.

[35] Howarth A.J., Peters A.W., Vermeulen N.A., Wang T.C., Hupp J.T., Farha O.K. (2017). Best Practices for the Synthesis, Activation, and Characterization of Metal–Organic Frameworks. Chem. Mater..

[36] Aijaz A., Karkamkar A., Choi Y.J., Tsumori N., Rönnebro E., Autrey T., Shioyama H., Xu Q. (2012). Immobilizing highly catalytically active Pt nanoparticles inside the pores of metal–organic framework: A double solvents approach. J. Am. Chem. Soc..

[37] Imperor-Clerc M., Bazin D., Appay M.-D., Beaunier P., Davidson A. (2004). Crystallization of β-MnO_2_ Nanowires in the Pores of SBA-15 Silicas:  In Situ Investigation Using Synchrotron Radiation. Chem. Mater..

[38] Shichibu Y., Negishi Y., Tsunoyama H., Kanehara M., Teranishi T., Tsukuda T. (2007). Extremely high stability of glutathionate-protected Au25 clusters against core etching. Small.

[39] Kawasaki H., Hamaguchi K., Osaka I., Arakawa R. (2011). ph-Dependent Synthesis of Pepsin-Mediated Gold Nanoclusters with Blue Green and Red Fluorescent Emission. Adv. Funct. Mater..

[40] Li L., Liu W., Chen R., Shang S., Zhang X., Wang H., Zhang H., Ye B., Xie Y. (2022). Atom-Economical Synthesis of Dimethyl Carbonate from CO_2_: Engineering Reactive Frustrated Lewis Pairs on Ceria with Vacancy Clusters. Angew. Chem. Int. Ed..

[41] Farrag M. (2021). In situ preparation of palladium nanoclusters in cerium metal-organic frameworks Ce-MOF-808, Ce-UiO-66 and Ce-BTC as nanoreactors for room temperature Suzuki cross-coupling reaction. Microporous Mesoporous Mater..

[42] Sun W., Li X., Sun C., Huang Z., Xu H., Shen W. (2019). Insights into the pyrolysis processes of Ce-MOFs for preparing highly active catalysts of toluene combustion. Catalysts.

[43] Cheng X.Q., Li S., Bao H., Yang X., Li Z., Zhang Y., Wang K., Ma J., Ullah A., Shao L. (2021). Poly (sodium-p-styrenesulfonate)-grafted UiO-66 composite membranes boosting highly efficient molecular separation for environmental remediation. Adv. Compos. Hybrid Mater..

[44] Stawowy M., Róziewicz M., Szczepańska E., Silvestre-Albero J., Zawadzki M., Musioł M., Łuzny R., Kaczmarczyk J., Trawczyński J., Łamacz A. (2019). The impact of synthesis method on the properties and CO_2_ sorption capacity of UiO-66 (Ce). Catalysts.

[45] Lu G.-P., Li X., Zhong L., Li S., Chen F. (2019). Ru@ UiO-66 (Ce) catalyzed acceptorless dehydrogenation of primary amines to nitriles: The roles of Lewis acid–base pairs in the reaction. Green Chem..

[46] Cavka J.H., Jakobsen S., Olsbye U., Guillou N., Lamberti C., Bordiga S., Lillerud K.P. (2008). A new zirconium inorganic building brick forming metal organic frameworks with exceptional stability. J. Am. Chem. Soc..

[47] Caratelli C., Hajek J., Cirujano F.G., Waroquier M., i Xamena F.X.L., Van Speybroeck V. (2017). Nature of active sites on UiO-66 and beneficial influence of water in the catalysis of Fischer esterification. J. Catal..

[48] Atzori C., Lomachenko K.A., Øien-Ødegaard S., Lamberti C., Stock N., Barolo C., Bonino F. (2019). Disclosing the Properties of a New Ce(III)-Based MOF: Ce_2_(NDC)_3_(DMF)_2_. Cryst. Growth Des..

[49] He J., Xu Y., Wang W., Hu B., Wang Z., Yang X., Wang Y., Yang L. (2020). Ce(III) nanocomposites by partial thermal decomposition of Ce-MOF for effective phosphate adsorption in a wide pH range. Chem. Eng. J..

[50] Bakuru V.R., Velaga B., Peela N.R., Kalidindi S.B. (2018). Hybridization of Pd Nanoparticles with UiO-66(Hf) Metal-Organic Framework and the Effect of Nanostructure on the Catalytic Properties. Chem.—A Eur. J..

[51] Zhai Y., Li Y., Hou Q., Zhang Y., Zhou E., Li H., Ai S. (2022). Highly sensitive colorimetric detection and effective adsorption of phosphate based on MOF-808 (Zr/Ce). New J. Chem..

[52] Cai X., Pan J., Tu G., Fu Y., Zhang F., Zhu W. (2018). Pd/UiO-66(Hf): A highly efficient heterogeneous catalyst for the hydrogenation of 2,3,5-trimethylbenzoquinone. Catal. Commun..

[53] Turner S., Lebedev O.I., Schröder F., Esken D., Fischer R.A., Tendeloo G.V. (2008). Direct Imaging of Loaded Metal−Organic Framework Materials (Metal@MOF-5). Chem. Mater..

[54] Zhang J., Cheng N., Ge B. (2022). Characterization of metal-organic frameworks by transmission electron microscopy. Adv. Phys. X.

[55] Liu L., Zhang D., Zhu Y., Han Y. (2020). Bulk and local structures of metal–organic frameworks unravelled by high-resolution electron microscopy. Commun. Chem..

[56] Zhang X., Hou F., Yang Y., Wang Y., Liu N., Chen D., Yang Y. (2017). A facile synthesis for cauliflower like CeO_2_ catalysts from Ce-BTC precursor and their catalytic performance for CO oxidation. Appl. Surf. Sci..

[57] Thommes M., Kaneko K., Neimark A.V., Olivier J.P., Rodriguez-Reinoso F., Rouquerol J., Sing K.S.W. (2015). Physisorption of gases, with special reference to the evaluation of surface area and pore size distribution (IUPAC Technical Report). Pure Appl. Chem..

[58] Lee Y., He G., Akey A.J., Si R., Flytzani-Stephanopoulos M., Herman I.P. (2011). Raman analysis of mode softening in nanoparticle CeO_2_− δ and Au-CeO_2_− δ during CO oxidation. J. Am. Chem. Soc..

[59] Huang P., Wu F., Zhu B., Gao X., Zhu H., Yan T., Huang W., Wu S., Song D. (2005). CeO_2_ nanorods and gold nanocrystals supported on CeO_2_ nanorods as catalyst. J. Phys. Chem. B.

[60] Xu Q., Kharas K.C., Datye A. (2003). The preparation of highly dispersed Au/Al_2_O_3_ by aqueous impregnation. Catal. Lett..

[61] Lopez-Sanchez J.A., Dimitratos N., Hammond C., Brett G.L., Kesavan L., White S., Miedziak P., Tiruvalam R., Jenkins R.L., Carley A.F. (2011). Facile removal of stabilizer-ligands from supported gold nanoparticles. Nat. Chem..

[62] Zhou Z., Kooi S., Flytzani-Stephanopoulos M., Saltsburg H. (2008). The role of the interface in CO oxidation on Au/CeO2 multilayer nanotowers. Adv. Funct. Mater..

[63] Camellone M.F., Fabris S. (2009). Reaction mechanisms for the CO oxidation on Au/CeO_2_ catalysts: Activity of substitutional Au^3+^/Au^+^ cations and deactivation of supported Au^+^ adatoms. J. Am. Chem. Soc..

[64] Abad A., Concepción P., Corma A., García H. (2005). A Collaborative Effect between Gold and a Support Induces the Selective Oxidation of Alcohols. Angew. Chem. Int. Ed..

[65] Crist V.B. (2000). Handbooks of Monochromatic XPS Spectra: The Elements and Native Oxides.

[66] Si R., Flytzani-Stephanopoulos M. (2008). Shape and crystal-plane effects of nanoscale ceria on the activity of Au-CeO_2_ catalysts for the water–gas shift reaction. Angew. Chem..

[67] Zhou Z., Flytzani-Stephanopoulos M., Saltsburg H. (2011). Decoration with ceria nanoparticles activates inert gold island/film surfaces for the CO oxidation reaction. J. Catal..

[68] Horváth A., Beck A., Stefler G.r., Benkó T., Sáfrán G.R., Varga Z., Gubicza J., Guczi L. (2011). Silica-supported Au nanoparticles decorated by CeO_2_: Formation, morphology, and CO oxidation activity. J. Phys. Chem. C.

[69] Casaletto M.P., Longo A., Martorana A., Prestianni A., Venezia A.M. (2006). XPS study of supported gold catalysts: The role of Au^0^ and Au^+δ^ species as active sites. Surf. Interface Anal..

[70] Li H., Jiao X., Li L., Zhao N., Xiao F., Wei W., Sun Y., Zhang B. (2015). Synthesis of glycerol carbonate by direct carbonylation of glycerol with CO_2_ over solid catalysts derived from Zn/Al/La and Zn/Al/La/M (M = Li, Mg and Zr) hydrotalcites. Catal. Sci. Technol..

[71] Shen Q., Yan H., Yuan X., Li R., Kong D., Zhang W., Zhang H., Liu Y., Chen X., Feng X. (2023). Tailoring morphology of MgO catalyst for the enhanced coupling reaction of CO_2_ and glycerol to glycerol carbonate. Fuel.

[72] Takeuchi K., Matsumoto K., Fukaya N., Sato K., Choi J.-C. (2022). Synthesis of Glycerol Carbonate from Glycerol and CO_2_ Using CaO as a Dehydrating Agent. Asian J. Org. Chem..

[73] Razali N., McGregor J. (2021). Improving product yield in the direct carboxylation of glycerol with CO_2_ through the tailored selection of dehydrating agents. Catalysts.

[74] Tomishige K., Gu Y., Nakagawa Y., Tamura M. (2020). Reaction of CO_2_ With Alcohols to Linear-, Cyclic-, and Poly-Carbonates Using CeO_2_-Based Catalysts. Front. Energy Res..

[75] Somorjai G.A., Park J.Y. (2008). Molecular Factors of Catalytic Selectivity. Angew. Chem. Int. Ed..

[76] Zhang M., Liu Q., Long H., Sun L., Murayama T., Qi C. (2021). Insights into Au nanoparticle size and chemical state of Au/ZSM-5 catalyst for catalytic cracking of n-octane to increase propylene production. J. Phys. Chem. C.

[77] Wang S., Wang J., Sun P., Xu L., Okoye P.U., Li S., Zhang L., Guo A., Zhang J., Zhang A. (2019). Disposable baby diapers waste derived catalyst for synthesizing glycerol carbonate by the transesterification of glycerol with dimethyl carbonate. J. Clean. Prod..

[78] Koranian P., Dalai A.K., Sammynaiken R. (2024). Production of glycerol carbonate from glycerol and carbon dioxide using metal oxide catalysts. Chem. Eng. Sci..

[79] Sheldon R.A. (2017). The E factor 25 years on: The rise of green chemistry and sustainability. Green Chem..

[80] Constable D.J., Curzons A.D., Cunningham V.L. (2002). Metrics to ‘green’chemistry—Which are the best?. Green Chem..

[81] Lim Y.N., Lee C., Jang H.-Y. (2014). Metal-Free Synthesis of Cyclic and Acyclic Carbonates from CO_2_ and Alcohols. Eur. J. Org. Chem..

[82] Song X., Wu Y., Pan D., Zhang J., Xu S., Gao L., Wei R., Xiao G. (2018). Functionalized DVB-based polymer catalysts for glycerol and CO_2_ catalytic conversion. J. CO2 Util..

[83] Liu H., Li Q.-Q., Zhou L., Deng B., Pan P.-H., Zhao S.-Y., Liu P., Wang Y.-Y., Li J.-L. (2023). Confinement of Organic Dyes in UiO-66-Type Metal–Organic Frameworks for the Enhanced Synthesis of [1,2,5]Thiadiazole[3,4-g]benzoimidazoles. J. Am. Chem. Soc..

[84] Melillo A., Cabrero-Antonino M., Ferrer B., Dhakshinamoorthy A., Baldoví H.G., Navalón S. (2023). MOF-on-MOF Composites with UiO-66-Based Materials as Photocatalysts for the Overall Water Splitting under Sunlight Irradiation. Energy Fuels.

