# Supplementary material for: Enhancing the Green Synthesis of Glycerol Carbonate: Carboxylation of Glycerol with CO2 Catalyzed by Metal Nanoparticles Encapsulated in Cerium Metal–Organic Frameworks
Source: Nanomaterials (Basel). 2024 Apr 9;14(8):650. doi: 10.3390/nano14080650 (PMC11055023; doi:10.3390/nano14080650)
Supplement: Supplementary file 1 [file nanomaterials-14-00650-s001.zip › nanomaterials-2940813-supplementary.pdf]

Supplementary Material

Enhancing the Green Synthesis of Glycerol Carbonate: Carboxylation of Glycerol with CO_2 Catalyzed by Metal Nanoparticles Encapsulated in Cerium Metal–Organic Frameworks

Simon Lukato, Michał Wójcik, Agnieszka Krogul-Sobczak and Grzegorz Litwinienko*

Faculty of Chemistry, University of Warsaw

Pasteura 1, 02-093 Warsaw, Poland

TABLE OF CONTENTS

Title	page
**Description of chemicals**	S-2
**Calculation of yield, conversion, TOF and TON**	S-2
**Table.S1.** ICP-MS data of 4%Au(H_2)@MOF1.	S-3
**Figure.S1.** SEM images showing small MOF nanoparticles less than 100 nm (A) and large octahedral particles surrounded by aggregates (small particles) (B).	S-4
**Figure S2.** FTIR of the synthesized MOF and catalysts materials.	S-4
**Figure S3.** Literature XRD patterns of two MOFs and their derivative	S-5
**Figure S4.** SEM image of 4%Au(H_2)@MOF1.	S-6
**Figure S5.** SEM-EDS elemental maps for 4%Au(H_2)@MOF1.	S-7
**Figure S6.** EDS elemental composition for 4%Au(H_2)@MOF1	S-8
**Figure S7.** Chromatogram of silylated GL and GC.	S-8
**Figure S8.** Survey XPS spectra of Au(H_2)@MOF1.	S-9
**References**	S-10

Chemicals

Ammonium Cerium(IV) nitrate (≥ 99.5 %), 1, 4-benzenedicarboxylic acid (BDC) (98 %, and sodium borohydride (98 %) were purchased from Sigma Aldrich, Poland. 1, 3, 5-benzenetricarboxylic acid (BTC) (≥ 98 %) were supplied by Chemat (Poland). Pyridine (99+ %), chlorotrimethylsilane (98 %) and Glycerol (99 %) were supplied by Thermo Scientific (Germany), polyethylenimine (PEI), Mw≈600 Da (Sigma Aldrich, Poland), and hydrogen tetrachloroaurate (≥99.9 %, Sigma Aldrich, Poland). 1, 8-diazabycyclo[5.4.0]undec-7-ene (98 %, Merk, Germany), 1, 1, 1, 3 ,3 ,3-Hexamethyldisilazane (≥98 %, ROTH). N-N-Dimethylformamide (99.8 %) and methanol (99.8 %) were supplied by POCH, Poland. Solvents were dried using activated molecular sieves before use and all other chemicals were used as supplied.

Crude GL was obtained from edible commercial rapeseed oil as follows: 250 mL of canola oil was pumped into the reaction using a peristaltic pump. 50 mL of 1M methanol solution of KOH was added using the same pump. The reactor was switched on to start the transesterification and stirred for 30 min. At the end of the transesterification, the resultant mixture was allowed to settle without agitation and two layers were obtained. The bottom layer (crude GL) was collected. It was dried over molecular sieves to ensure complete exclusion of any water content. Estimated quantification based on comparison with moles of GL per mL of >99% pure commercial GL found the crude GL fraction to be about 48-54% GL after simple drying.

Calculation of conversion, yield, TOF and TON

The calculations were performed using the following equations:

$$\textbf{Conversion}\ [\%] = \frac{(n_{GL(start)} - n_{GL(final)})}{n_{GL(start)}} \times 100\% \qquad \text{(eq. S1)}$$

$$\textbf{Yield}\ [\%] = \frac{n_{GLC(final)}}{n_{GL(start)}} \times 100\% \qquad \text{(eq. S2)}$$

where $n_{GL(start)}$ is starting amount of GL (in mmoles), $n_{GL(final)}$ – mmoles of GL found in post reaction mixture, $n_{GLC(final)}$ – mmoles of GLC found in post reaction mixture, $n_{GL(converted)}$ - mmoles of GL converted.

Taking into account very low conversion (14%, see Table 2, run 6) for the pristine MOF (without Au) observed after 25 h it can be concluded that there is very small (negligible) contribution of catalytic activity of the MOF without embedded Au, and Turnover Numbe (TON) and Turnover Frequency

(TOF) parameters were exclusively calculated based on moles of Au as an active component, and the amount of GL converted):

$$\mathbf{TON}_{\mathrm{GL}} = \frac{n_{GL(converted)}}{n_{Au}} = \frac{n_{GL(start)} - n_{GL(final)}}{n_{Au}} \quad \text{(eq. S3)}$$

$$\mathbf{TOF}_{\mathrm{GL}}\ [\mathrm{h}^{-1}] = \frac{\mathbf{TON}_{\mathrm{GL}}}{reaction\ time\ (in\ \mathrm{hours})} \quad \text{(eq. S4)}$$

or basing on the produced GLC:

$$\mathbf{TON}_{\mathrm{GLC}} = \frac{n_{GLC(final)}}{n_{Au}} \quad \text{(eq. S5)}$$

$$\mathbf{TOF}_{\mathrm{GLC}}\ [\mathrm{h}^{-1}] = \frac{\mathbf{TON}_{\mathrm{GLC}}}{reaction\ time\ (in\ \mathrm{hours})} \quad \text{(eq. S6)}$$

where n_{Au} is the estimated amount of Au (in mmoles) in 50 mg of the whole catalyst (Au + MOF). Amount of Au in the catalyst was determined using ICP-MS results, and mass of Au was 1.25 mg (n_{Au}= 0.0064 mmol).

Using eq. S3 and S4, with $n_{GL\mathrm{(converted)}}$= 52% x 12.5 mmol = 6.5 mmol and for reaction time 13 hours, **$\mathrm{TON}_{\mathrm{GL}}$ is 6.5 mmol/0.0064 mmol = 1016, and $\mathrm{TOF}_{\mathrm{GL}}$ = 1016/13 hours = 78 h^{-1}.**

The same calculations with eq. S5 and S6, with $n_{\mathrm{GLC(final)}}$= 44% x 12.5 mmol = 5.5 mmol gave **$\mathrm{TON}_{\mathrm{GLC}}$ = 5.5/0.0064 = 859, and $\mathrm{TOF}_{\mathrm{GLC}}$ = 66 hr^{-1}.**

Our parameters can be compared with the results described in the literature. For example, Kulal *et al.*[1] used Zn/CeO_2 catalyst (molar fraction x_{Zn}= 0.105), and TON and TOF values calculated from the results presented by these authors in Table S5, 0.99 mmol of catalyst caused 90.4% conversion of 10 mmol of GL during 1.8 hour (ie., 9 mmol of GL was converted) , therefore, $\mathrm{TON}_{\mathrm{GL}}$ = 9mmol/0.99mmol ≈ 9, and TOF = 9 /5 = 1.8 h^{-1}. TON and TOF values based on the results obtained by Liu et al. [2] for 1.72 g of CeO_2 catalyst (10mmol) and with $n_{\mathrm{GLC(final)}}$=7.9 mmol (yield=78.9% after 5 hours) obtained $\mathrm{TON}_{\mathrm{GLC}}$ = 7.9mmol/10mmol = 0.79, and $\mathrm{TOF}_{\mathrm{GLC}}$ = 0.79/5h = 0.16 h^{-1} .

Table S1. ICP-MS results of the $Au(H_2)$@MOF1 catalyst

**Catalyst**	**mg of Au/20 mg catalyst**	**mg of Ce /20 mg of catalyst**
Fresh catalyst	0.501	10.768
Spent catalyst	0.344	8.805

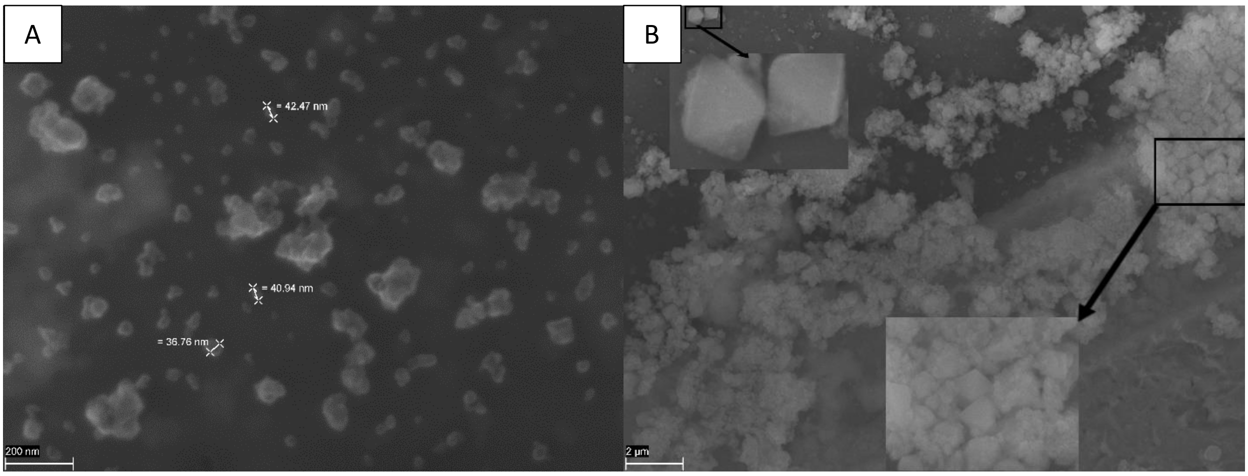

Figure S1. SEM images of the pure MOF showing. A) An area with many small MOF nanoparticles less than 100 nm. B) Large octahedral particles surrounded by aggregates (small particles).

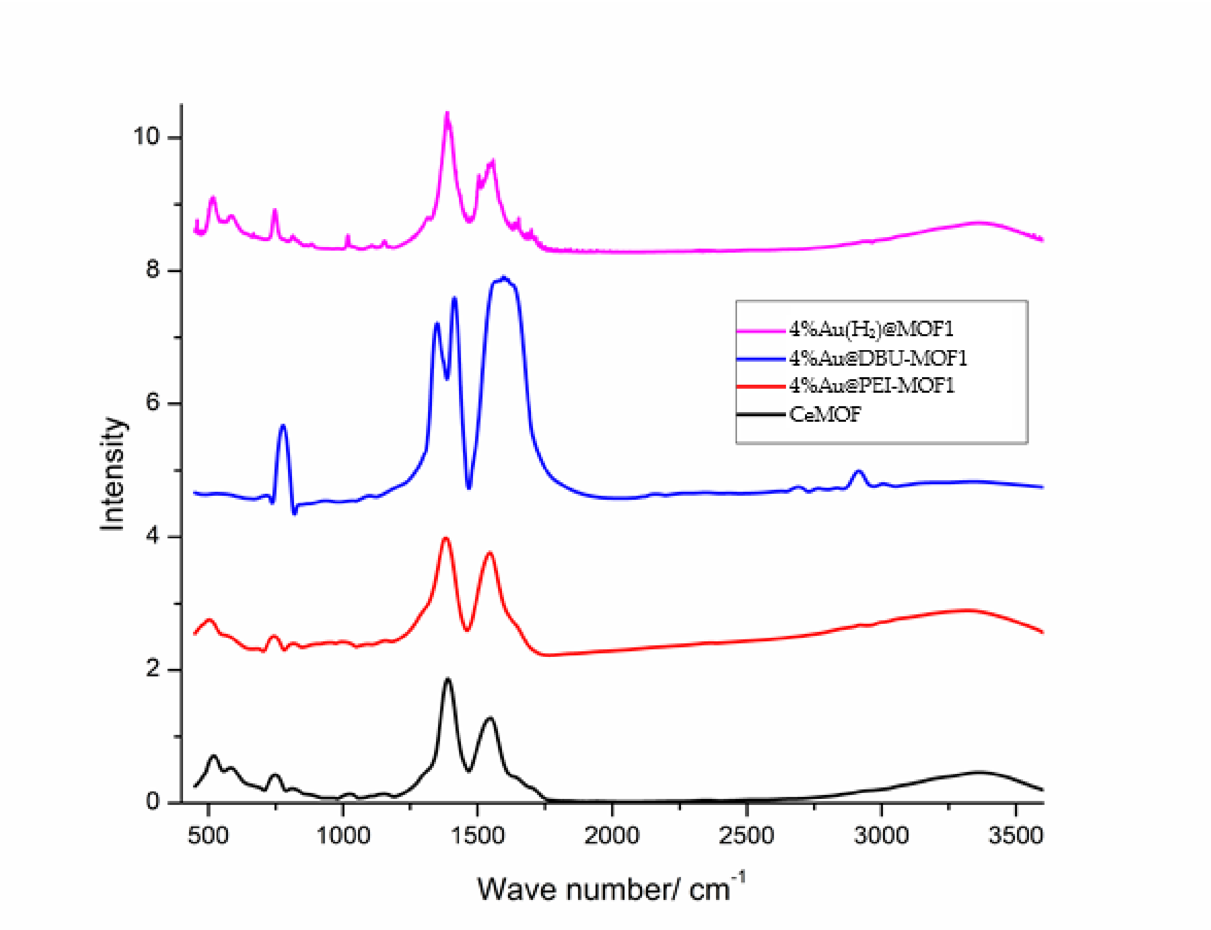

Figure S2. FTIR of the synthesized materials.

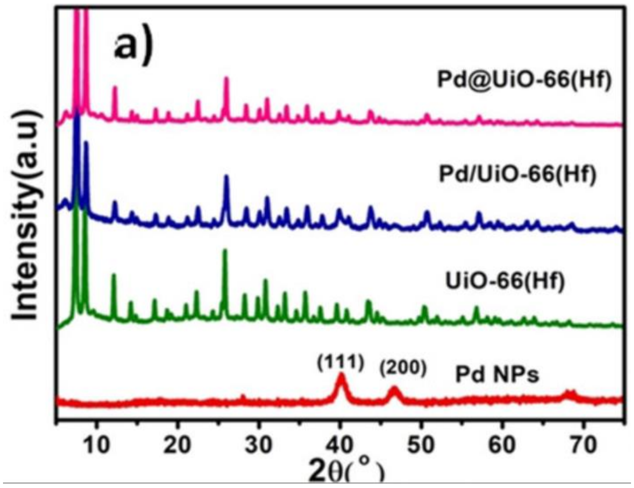

Figure S3A.
Literature XRD for UiO-66 (Hf) and Pd/UiO-66(Hf) Pd@UiO-66(Hf).[3]
Reproduced with permission Copyright Wiley-VCH GmbH.

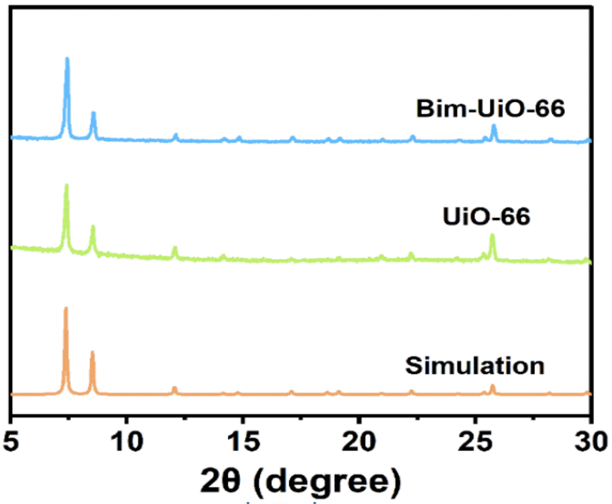

Figure S3B.
Literature XRD for UiO-66 (Zr).[4]
Reproduced with permission-Copyright: ACS.

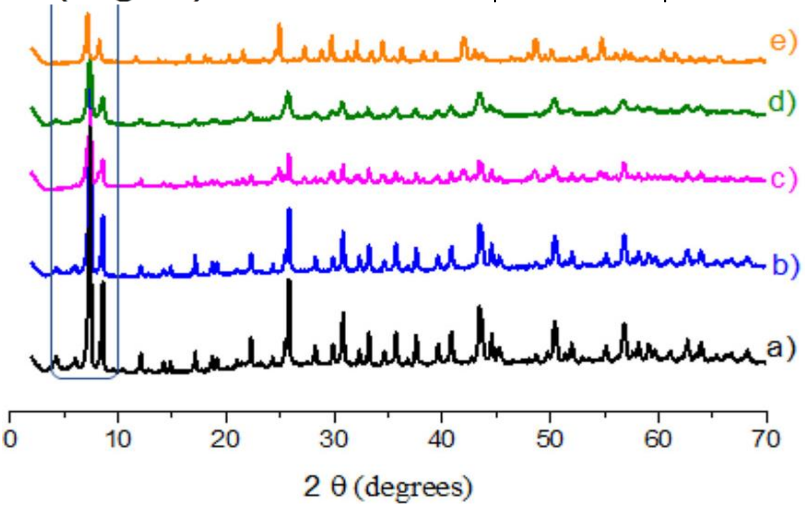

Figure S3C.
PXRD patterns of UiO-66-(Zr)-NH2 (a), UiO-66(Zr)-NH2-200@UiO-66(Ce) (b), UiO-66(Zr)-NH2-25@UiO-66(Ce) (c), UiO-66(Ce)-200@UiO-66(Zr)-NH2 (d), and UiO-66(Ce) (e). The inset shows a zoom-in view of the low-angle region.[5] Reproduced with permission. Copyrights ACS.

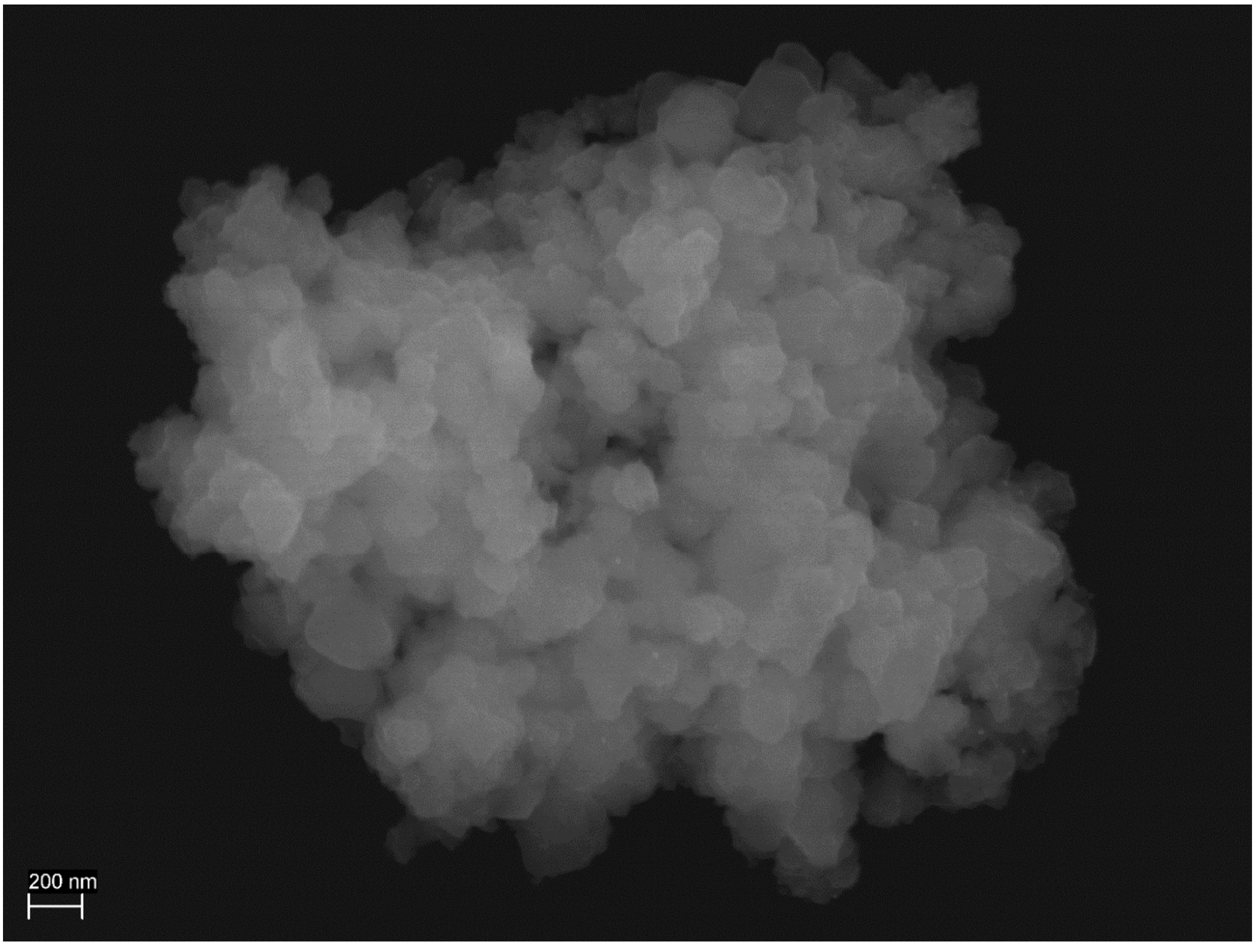

Figure S4. SEM image of 4%$Au(H_2)$@MOF1.

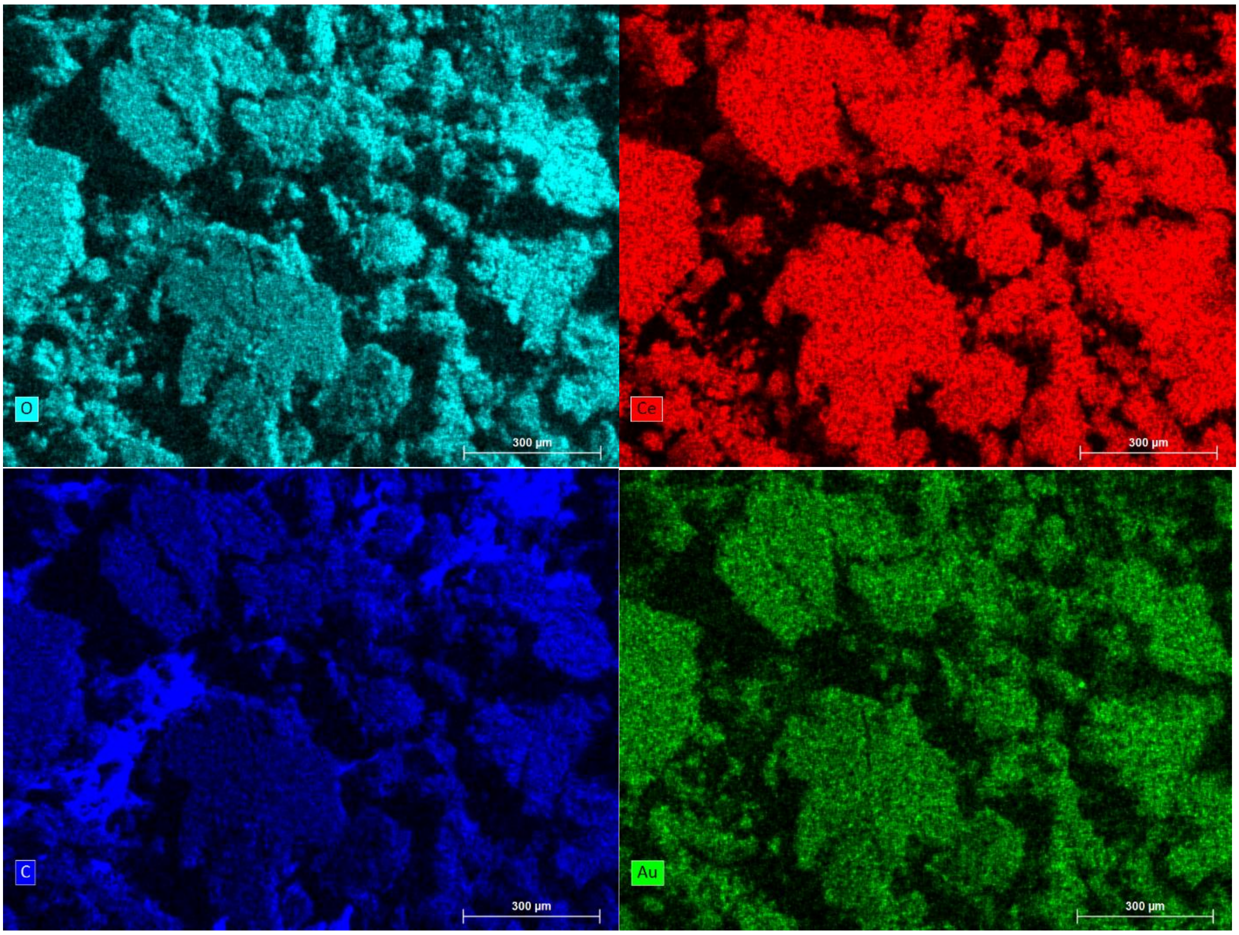

Figure S5. SEM EDS elemental mapping of 4%Au(H_2)@MOF1.

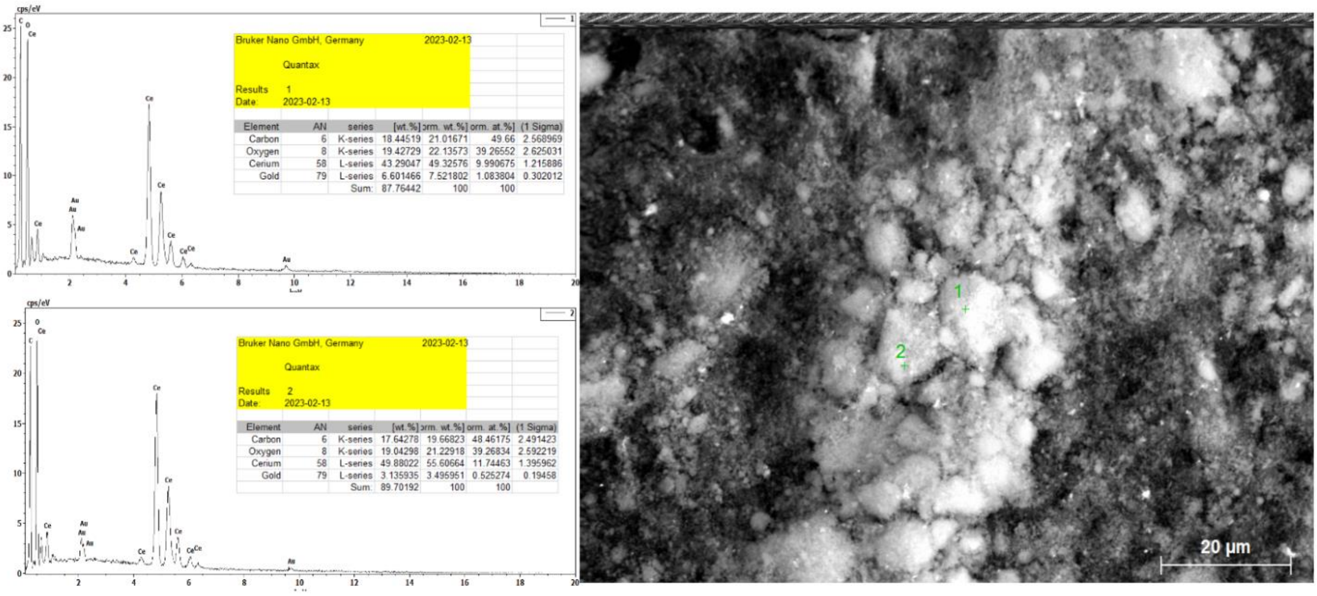

Figure S6. EDS elemental composition for 4%Au(H_2)@MOF1.

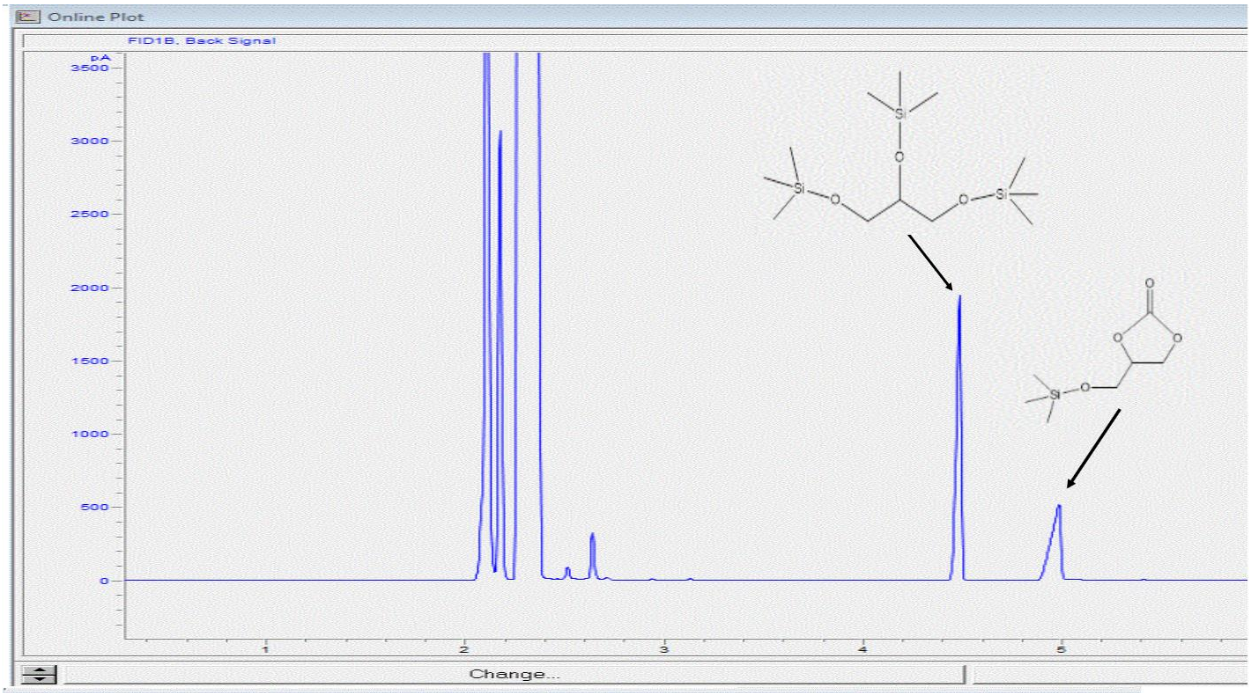

Figure S7. Chromatogram of silylated GL and GC. The peak of the internal standard (cumene) is at about 2.7 min.

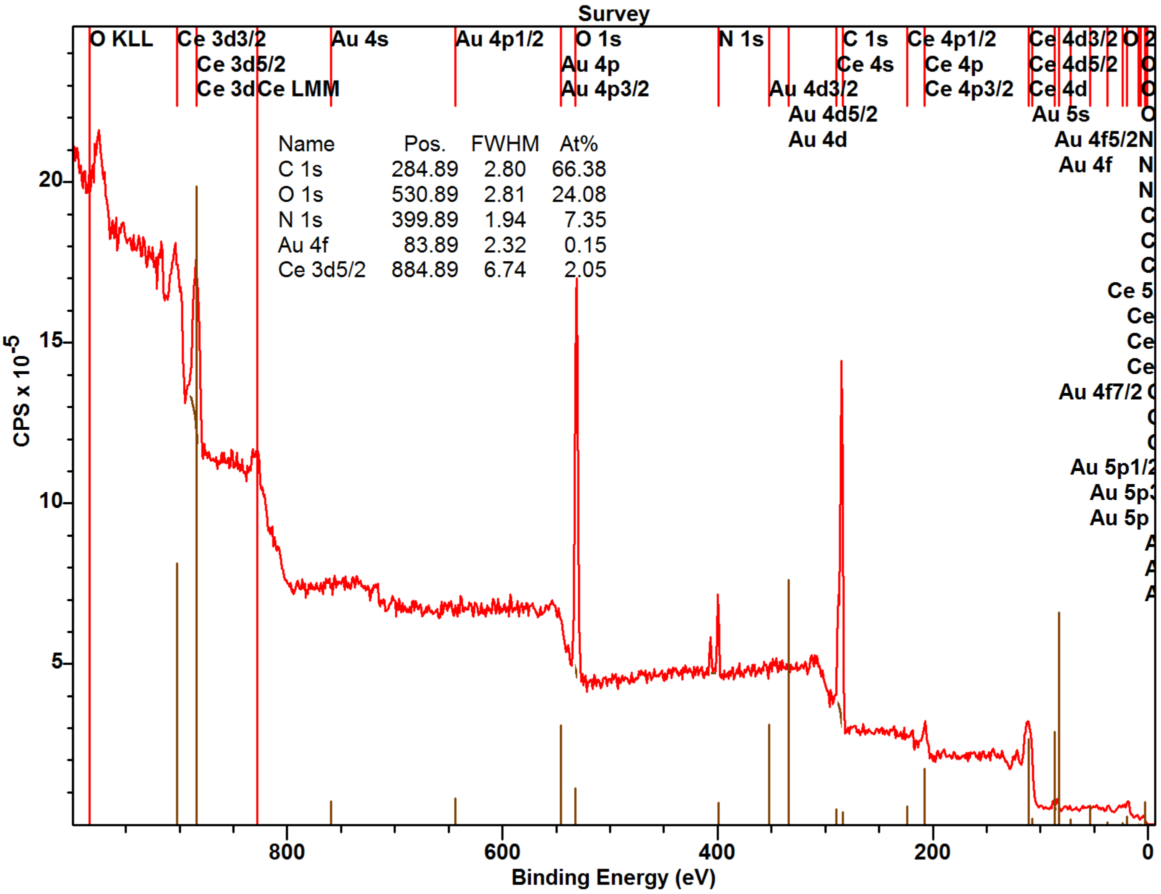

Figure S8. Survey XPS spectra of Au(H_2)@MOF1.

REFERENCES

1. Kulal, N.; Vetrivel, R.; Ganesh Krishna, N. S.; Shanbhag, G. V., Zn-Doped CeO2 Nanorods for Glycerol Carbonylation with CO2. *ACS Applied Nano Materials* **2021,** *4* (5), 4388-4397.
2. Liu, J.; Li, Y.; Zhang, J.; He, D., Glycerol carbonylation with CO2 to glycerol carbonate over CeO2 catalyst and the influence of CeO2 preparation methods and reaction parameters. *Applied Catalysis A: General* **2016,** *513*, 9-18.
3. Bakuru, V. R.; Velaga, B.; Peela, N. R.; Kalidindi, S. B., Hybridization of Pd Nanoparticles with UiO-66(Hf) Metal-Organic Framework and the Effect of Nanostructure on the Catalytic Properties. *Chemistry – A European Journal* **2018,** *24* (60), 15978-15982.
4. Liu, H.; Li, Q.-Q.; Zhou, L.; Deng, B.; Pan, P.-H.; Zhao, S.-Y.; Liu, P.; Wang, Y.-Y.; Li, J.-L., Confinement of Organic Dyes in UiO-66-Type Metal–Organic Frameworks for the Enhanced Synthesis of [1,2,5]Thiadiazole[3,4-g]benzoimidazoles. *Journal of the American Chemical Society* **2023,** *145* (32), 17588-17596.
5. Melillo, A.; Cabrero-Antonino, M.; Ferrer, B.; Dhakshinamoorthy, A.; Baldoví, H. G.; Navalón, S., MOF-on-MOF Composites with UiO-66-Based Materials as Photocatalysts for the Overall Water Splitting under Sunlight Irradiation. *Energy & Fuels* **2023,** *37* (7), 5457-5468.
